# Trade‐Offs Between Growth, Longevity, and Storage Carbohydrates in Herbs and Shrubs: Evidence for Active Carbon Allocation Strategies

**DOI:** 10.1111/pce.15444

**Published:** 2025-02-27

**Authors:** Kenz Raouf Samraoui, Adam Klimeš, Veronika Jandová, Nela Altmanová, Jan Altman, Miroslav Dvorský, Vojtech Lanta, Klára Řeháková, Adam Taylor Ruka, Pavel Fibich, Pierre Liancourt, Jiří Doležal

**Affiliations:** ^1^ Institute of Botany of the Czech Academy of Sciences Průhonice Czech Republic; ^2^ Faculty of Science University of South Bohemia České Budějovice Czech Republic; ^3^ Faculty of Mathematics and Natural Sciences, Department of Biological Sciences University of Bergen Bergen Norway; ^4^ State Museum of Natural History Stuttgart Stuttgart Germany

**Keywords:** active accumulation, carbon allocation strategies, carbon allocation trade‐offs, longevity, nonstructural carbohydrates, plant growth

## Abstract

Plants store nonstructural carbohydrates (NSCs) like starch, fructans and soluble sugars to support metabolism, stress tolerance and defence during low photosynthesis, ultimately influencing their growth and longevity. However, the relationship between NSC composition and growth or persistence in wild plants remains unclear. This study explores trade‐offs between growth, longevity and NSCs in 201 plant species across diverse climates in the Western USA, spanning 500–4300 m in elevation and 80–1000 mm in precipitation. Annual growth rates and plant ages were derived from the ring widths of semidesert, steppe and alpine herbs and shrubs, along with NSC profiles in their roots and rhizomes. Results showed an inverse relationship between growth and age, with total NSC, starch and fructan levels negatively correlated with growth, supporting the growth‐longevity and growth‐storage trade‐off hypotheses. Conversely, higher growth rates were linked to soluble sugars, suggesting that climate‐driven growth limitations alone do not explain increased NSCs. Fructans were positively associated with longevity, especially in long‐lived desert shrubs and alpine herbs, underscoring NSCs' active role in survival strategies. These findings challenge the carbon surplus hypothesis, suggesting that plants actively use specific NSCs to balance growth and persistence, with energy‐rich sugars promoting growth and osmoprotective fructans enhancing longevity.

## Summary statement

Plants store nonstructural carbohydrates (NSCs) for survival, but their link to growth and longevity remains unclear. This study of 201 species found higher starch and fructan levels correlate with slower growth and greater longevity, while soluble sugars promote faster growth, supporting trade‐offs between storage, growth and lifespan.

## 1 Introduction

The allocation of carbon assimilates between structural growth and various storage compounds shapes plant life strategies, competitiveness and longevity (Mooney 1972; Hartmann and Trumbore 2016). Plants store significant amounts of nonstructural carbohydrates (NSCs), such as starch, fructans and soluble sugars (Van den Ende and Oner 2023), which serve various functions: they act as energy reserves during low photosynthesis or nutrient scarcity (Guo et al. 2020; Hartmann et al. 2020), support respiration and biochemical reactions (Couée et al. 2006; Blumstein et al. 2023), and enhance stress tolerance by stabilising cells under drought or freezing (Rosas et al. 2013; Chlumská et al. 2022). NSCs also contribute to defence by converting to secondary metabolites that deter herbivores and pathogens (Herms and Mattson 1992; Huang et al. 2019) and support growth by providing energy for cell division and elongation. NSCs play a central role in balancing plant growth and storage, ultimately affecting their survival and longevity (Holland et al. 2019). However, the trade‐offs between total and specific NSC compounds in relation to growth rate and persistence remain poorly understood, particularly in wild herbaceous plants (Agrawal 2020; Lundgren and Des Marais 2020).

NSCs serve as dynamic carbon reservoirs, modulated by the balance between carbon supply from photosynthesis and demand for growth (source‐sink dynamics; Hartmann and Trumbore 2016). Slow‐growing, long‐lived plants generally prioritise starch and fructan storage over rapid growth to enhance stress tolerance and survival in challenging conditions, consistent with the growth‐storage and growth‐longevity trade‐off hypotheses (Arendt 1997; Black et al. 2008). In contrast, fast‐growing, short‐lived species tend to favour energy‐rich soluble sugars, enabling quick growth or recovery from disturbances like frost damage or herbivory (Hartmann and Trumbore 2016; Smith and Stitt 2007; Sulpice et al. 2009). This suggests that carbon allocation among NSC types may be linked to variations in growth rates and lifespan across species (Smith and Stitt 2007; Sulpice et al. 2009; Hartmann and Trumbore 2016). However, empirical evidence across diverse species and environments remains limited.

Understanding the role of NSCs in plant growth and persistence, particularly among wild herbaceous species, is limited due to a lack of data on annual growth increments, plant age, and their NSCs stored in belowground organs (Smith and Stitt 2007; Sala et al. 2012). Recent advances in herbchronology have enabled the examination of annual growth rings in perennial dicot forbs, shedding light on their age and growth history (Gärtner and Schweingruber 2013; Doležal et al. 2018; Chondol et al. 2023). Additionally, the allocation of carbohydrates across different environments, plant families and growth forms remains underexplored (Dietze et al. 2014; González‐Paleo and Ravetta 2015; Chlumská et al. 2022). To elucidate the relationship between NSCs and plant growth, it is crucial to consider environmental variations (Chapin et al. 1990), phylogenetic relationships (Adams and Collyer 2017), and growth forms (Hiltbrunner et al. 2021). Although some studies have associated NSC levels with growth (Sala et al. 2012; Dietze et al. 2014; Huang et al. 2019; Blumstein et al. 2023), comprehensive interspecific research encompassing diverse growth forms beyond commonly studied trees remains limited, especially in challenging environments like deserts and high mountains.

It still remains unresolved whether NSCs are actively stored to support plant growth and survival during carbon stress or if their accumulation is merely a passive consequence of growth limitations (Wiley et al. 2013; Dietze et al. 2014). Under harsh conditions, plants may prioritise NSC accumulation to enhance survival, emphasising persistence over rapid growth (Körner 2003; Chlumská et al. 2022). This selective carbon allocation aligns with theories suggesting environmental constraints lead to accumulating NSCs, either passively or through adaptive adjustments (Prescott et al. 2020; Wiley and Helliker 2012). According to the carbon surplus hypothesis, limitations on growth (particularly in meristematic activity) may drive NSC accumulation under harsh conditions more than carbon availability from photosynthesis. Meristematic cambial processes, which are critical for growth, are typically more affected by cold or drought than photosynthesis (Chapin et al. 1990), leading to increased overall NSC content (Prescott et al. 2020; Zepeda et al. 2022). However, plants may favour specific carbon assimilates—like fructans or simple sugars—over total NSCs to survive in harsh settings (Chlumská et al. 2022; Van den Ende and Oner 2023). This strategy of accumulating targeted carbohydrates for long‐term reserves enhances resilience during prolonged dormancy or nutrient scarcity, potentially extending lifespan (Körner 2003; Suprasanna et al. 2016). Plants generally remobilise complex carbohydrate compounds, such as starch, under challenging environmental conditions into simpler sugars to provide energy (Martínez‐Vilalta et al. 2016). Starch is a main storage carbohydrate, sustaining growth during resource scarcity (Chapin et al. 1990; Hartmann and Trumbore 2016). Conversely, soluble sugars like glucose and fructose act as energy reserves for rapid growth in favourable conditions (Martínez‐Vilalta et al. 2016). Fructans, another NSC compound, contribute to osmoprotection, aiding plants in cold environments by preserving cellular integrity during freezing events (Livingston et al. 2009; Van den Ende 2013). Thus, investing in specific rather than total NSCs may reflect a life strategy prioritising persistence and longevity over rapid growth (Sala et al. 2012; Blumstein et al. 2022).

This study investigates the trade‐offs between growth, persistence and NSCs reserves across more than 200 vascular plant species in the diverse climatic zones of the Western United States. This region encompasses various climates that significantly influence plant growth and physiology (Tilman 1988; Chapin et al. 1990; Dietze et al. 2014). The study spans a wide climatic range from the Sonoran and Mojave deserts to the cold northern Rockies, with elevations from 500 to 4300 m a.s.l., annual precipitation from 80 to 1000 mm, and temperatures ranging from −4°C to 25°C. We investigate whether a negative correlation exists between total NSCs and interspecific growth rates, assess how different NSC compounds affect growth and longevity (persistence ability indicated by the age of the largest and possibly oldest plants sampled), and contribute to the discussion on whether NSCs accumulate passively or actively support growth and longevity.

Initially, we assess how environmental conditions, growth forms and phylogeny contribute to variations in radial growth derived from the ring widths on the oldest plant parts, plant age and NSCs among species. After accounting for these confounding factors, we focus on three main areas: (1) exploring the potential negative correlation between total NSCs and interspecific growth rates (the growth‐storage trade‐off hypothesis), (2) evaluating how different NSC compounds (starch, fructans, simple sugars) affect annual growth rates and plant age (the growth‐longevity trade‐off hypothesis) and (3) investigating whether carbohydrates accumulate passively due to growth limitations or actively contribute to growth and longevity (the carbon surplus hypothesis). We hypothesise that an inverse correlation between total NSCs and growth, combined with no significant relationship between NSC compounds and growth or longevity, suggests that NSCs function as an energy reservoir during constrained meristematic processes, thus supporting the carbon surplus hypothesis (Prescott et al. 2020; Zepeda et al. 2022). Conversely, if we find no correlation between growth and total NSCs but significant associations between specific NSC compounds and growth, temporal growth variation and longevity, this could challenge the carbon surplus hypothesis. Such a scenario would indicate that plants actively utilise different NSC types for defence or rapid energy rather than simply accumulating them, aligning more closely with the growth‐longevity trade‐off hypothesis (Sala et al. 2012; Blumstein et al. 2022).

## 2 Material and Methods

### 2.1 Study Area and Sampling Locations

**Figure 1:**
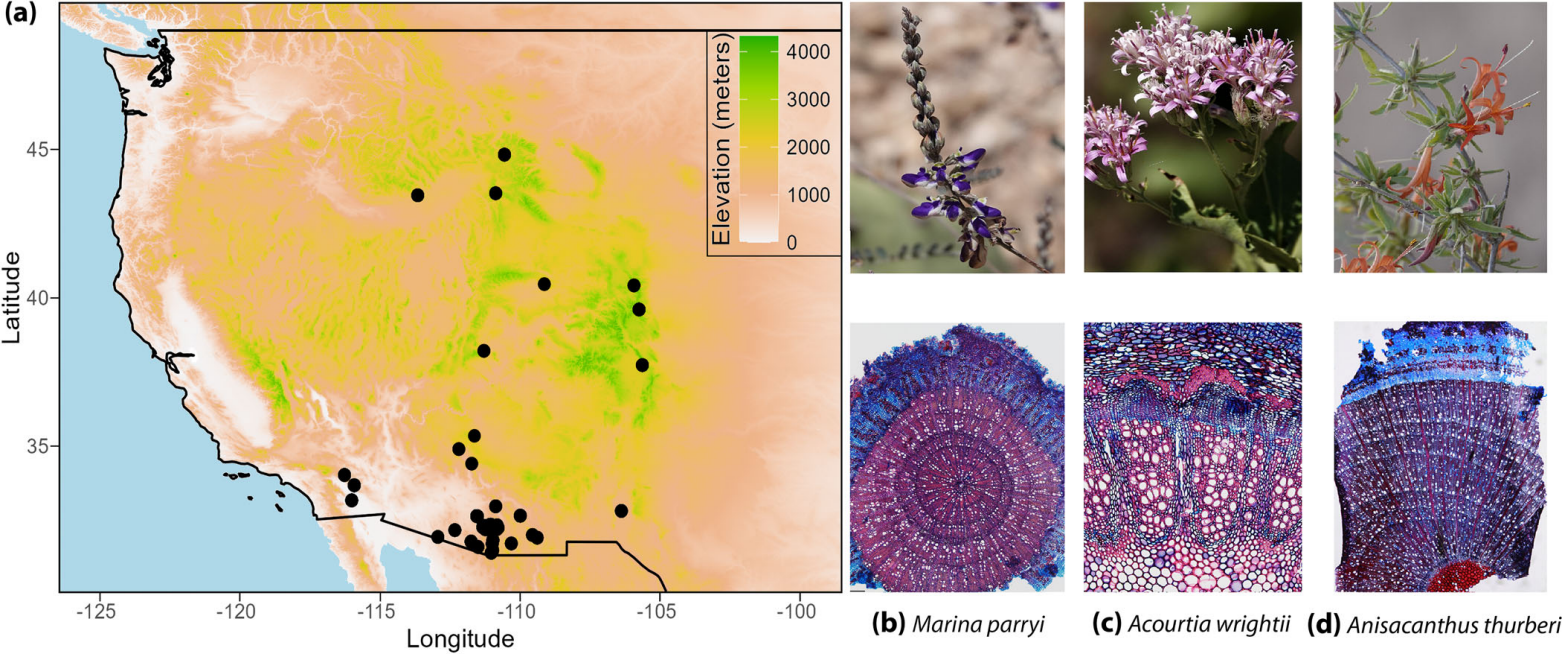
(a) Elevation map depicting the study area in the Western United States, with sampled locations denoted by black dots. (b–d) Examples of studied species and their double‐stained cross‐sections of root collars with growth rings.

This study focuses on 201 dicot vascular plant species (562 individual plants) belonging to 49 families collected from 43 sites in seven states (Arizona, California, Colorado, Idaho, New Mexico, Utah and Wyoming) of the Western United States during 2018–2019 (Figure 1, Supporting Information Table S1). The species selection was based on their ecological and geographical prevalence, availability across the sampled eco‐regions, and their representation of diverse habitats, growth forms and life histories. The Western United States encompasses diverse bioclimatic zones shaped by distinct temperature and precipitation regimes related to varied geomorphological features and orographic barriers (McLaughlin 1989; Seager et al. 2018). The study spanned a wide climatic range, from the northern cold Rockies to the desertic Sonoran and Mojave in the south. The elevation ranged from 533 m a.s.l. in Organ Pipe National Park to 4313 m a.s.l. in the Colorado Rockies. Total annual precipitation exhibited significant variability across the region, with measurements ranging from < 200 mm in the southwestern areas to > 2300 mm in the northeastern reaches. Furthermore, mean annual temperatures displayed an equally wide gradient, ranging from −5°C to 25°C, reflecting the contrasting thermal regimes experienced within the area.

### 2.2 Anatomical Growth and Age Analyses

We sampled five common plant species at each study site, selecting three of the largest individuals per species. We assumed they were among the oldest in the population and representative of each species' maximum age and growth longevity under local conditions. To fully understand how total and individual NSC types relate to plant persistence, we sampled plants across a spectrum of lifespans, from short‐lived to long‐lived species. Short‐lived herbs (1–2 years old) represent only a fraction of the diversity in plant life histories and strategies. While examining NSC storage in these species can provide valuable insights into the role of carbohydrates in supporting rapid growth and reproduction, they may not fully capture the dynamics and functions of NSCs in long‐lived plants. Among the 201 species, 70 were short‐lived herbs (1–2 years old), 112 perennial herbaceous species, and 19 woody perennials, mostly small shrubs. Plants were collected at full maturity during their peak growing seasons, from March to May for semidesert species and from May to August for steppe and alpine species at higher elevations.

Each short‐lived herb and perennial forb was carefully excavated from the soil and separated into belowground parts (roots, rhizomes) and aboveground parts (stems, leaves). The rhizospheric soil was removed by shaking and washing, and the plant materials were stored in paper bags. A 5 cm segment was taken from the oldest plant parts, such as the root collar in taprooted forbs or the oldest rhizome section in clonal perennials. For woody perennials, a 2 cm disc was cut from the base of the largest stem. These samples were preserved in 40% ethanol to keep the tissue soft and prevent mold growth. Plant age and growth data were obtained following standard protocols (Gärtner and Schweingruber 2013; Doležal et al. 2018). In the lab, using a sledge microtome, we cut cross‐sections of the oldest tissue, stained them with Astra Blue and Safranin, and mounted them with Canada balsam (Doležal et al. 2018). Annual rings were counted in the root collar zone, a transition zone between stem and root, typically preserved in perennial dicots with main taproots (Büntgen et al. 2015) or the oldest rhizome parts in clonal plants (Klimešová et al. 2019). We captured high‐resolution images using an Olympus BX53 microscope and DP73 camera with cellSense Entry 1.9 software to count rings and measure their increment width. Annual rings were generally well developed in the studied plants from hot semideserts, dry sagebrush steppes and cold mountains due to the prevalence of taproots over rhizomes (Supporting Information Table S2) and their predominantly ring‐porous and semi‐ring‐porous xylem with distinct earlywood and latewood (Doležal et al. 2018). We know that clonal plants tend to discard older tissues and allocate resources in favour of new growth (Chondol et al. 2023), making the age of existing active (live) tissue in clonal plants younger than the potential age of vegetatively reproducing genet (age based on the time elapsed since zygote formation), which may be tens to hundreds of years in clonal plants (Klimešová et al. 2019). We calculated mean radial growth rates and growth variability (coefficient of variation, CV) from annual ring widths measured along two radii per cross‐section, with age estimates based on the maximum ring count. The age of the largest, likely oldest individuals served as a proxy for maximum lifespan (hereafter referred to as longevity or persistence). It was analysed alongside NSC levels as predictors of longevity.

### 2.3 Environmental Variables

To investigate how environmental factors, specifically temperature and precipitation, influence our targeted variables, we used mean annual temperature and precipitation data from the CHELSA Bioclim data set (Karger et al. 2017), which includes a comprehensive range of bioclimatic variables derived from monthly mean, maximum and minimum temperature, as well as mean precipitation values. We employed Bio1 and Bio12 for our analyses, representing mean annual air temperature (mean annual daily air temperatures averaged over 1 year) and annual precipitation amount (accumulated over 1 year), respectively. The data set had a 1/24 degree spatial resolution and covered the period from 1901 to 2021, forming a robust foundation to explore the relationships between environmental factors and the variables under investigation.

### 2.4 Starch and Fructan Analyses

Immediately after collection, the samples from the plant individuals designated for NSC analysis were oven‐dried to a constant weight. Carbohydrate analysis focused on belowground rhizome and root organs (e.g., Janeček et al. 2015; Landhäusser et al. 2018). Total NSC values were calculated by summing all carbohydrate types in each sample and expressed as a percentage of the dry mass of the storage organ. For starch analysis, approximately 100 mg of the sample was extracted in 80% ethanol at 83°C for 12 min in a water bath, followed by centrifugation at 3000 rpm for 10 min. The supernatant was strained, and this extraction process was repeated three times before being set aside for future analysis. The starch in the pellet remaining after ethanol extraction was enzymatically hydrolysed using thermostable α‐amylase at 100°C until fully dissolved. Starch dextrins were then converted to glucose using amyloglucosidase at 50°C. The glucose concentration was measured spectrophotometrically after adding glucose oxidase/peroxidase (GOPOD) reagent, forming a quinoneimine dye.

The fructan content was analysed using an initial step to remove galactosyl sucrose oligosaccharides (raffinose family oligosaccharides, RFOs), following methods from previous studies (Chlumská et al. 2022). This involved specific hydrolysis of fructans with fructanase. To extract the fructans, approximately 100 mg of the sample was boiled in 25 ml of distilled water for 15 min while stirring. The suspension was then cooled and filtered through paper containing particles smaller than 11 µm (Whatman, grade 1). In the first step, 0.2 mL of the filtered solution was treated with 50 µL of α‐galactosidase (200 U/mL in 50 mM sodium acetate buffer at pH 4.5) for 30 min at 40°C to remove all RFOs. Next, sucrase, β‐amylase, pullulanase and maltase were added, and the solution was incubated at 30°C to eliminate other poly‐, di‐ and monosaccharides. The third step involved adding alkaline borohydride at 40°C to reduce the products (glucose and fructose) from the previous reactions into sugar alcohols, which were then removed by adding acetic acid. Finally, the fructans and fructooligosaccharides (FOS) reduced by borohydride were hydrolysed by exo‐inulinases, endo‐inulinases, and endo‐levanase at 40°C. The glucose and fructose produced during enzymatic hydrolysis were reduced using PAHBAH solution (p‐hydroxybenzoic acid hydrazide), and the resulting colour complex was measured spectrophotometrically.

### 2.5 Carbohydrates Soluble in Ethanol (Simple or Soluble Sugars) Analysis

The supernatant from the earlier analysis (as described in the starch analysis) was dried at 50°C and then redissolved in 10 ml of distilled water. It was shaken for half a day and subsequently filtered through nitrocellulose nitrate membrane filters with a particle retention threshold greater than 0.4 μm (Pragopor 6). The resulting filtrate was transferred to vials and subjected to analysis for ethanol‐soluble carbohydrates (including sugar alcohols, glucose, galactose, fructose, sucrose, raffinose and verbascose) using ion exchange chromatography (HPAEC‐PAD). An ion exchange chromatography system with a nonlinear elution gradient of distilled water and 5–225 mM NaOH was employed. The analysis of the species samples was carried out using the Dionex ICS‐3000, utilising the CarboPac PA10 column with an isocratic elution profile involving 18 mM NaOH at a flow rate of 1 mL/min and a temperature of 40°C. The chromatography program duration lasted approximately 65 min.

### 2.6 Data Analyses

First, we examined relationships among variables within three groups: environmental conditions, NSCs, growth (mean and CV) and longevity using a standardised major axis for all variable pairs and tested their correlations. Second, we investigated the effect of NSCs on growth and longevity, environmental conditions on NSCs, and environmental conditions on growth and longevity using phylogenetic linear models (Freckleton et al. 2002). To do so, we assessed the effects of all variables within the predictor group on the response group variables. To discern direct effects of environmental conditions' and NSCs' influence on growth and longevity, two models were employed: one with solely environmental conditions or NSCs as predictors, and another with both environmental conditions and NSCs as predictors. This assessment encompassed running the models using total NSCs and NSCs' individual compounds ‐ starch, fructans and simple sugars. F tests with type II sum of squares were used to evaluate predictor effects in all models. All models included growth form as a covariate. Before analysis, we transformed growth, longevity and total NSCs, starch fructans and simple sugars dry content using natural logarithm. In all models, we estimated phylogenetic signal strength (Pagel's λ; Pagel 1999) through maximum likelihood.

**Figure 2:**
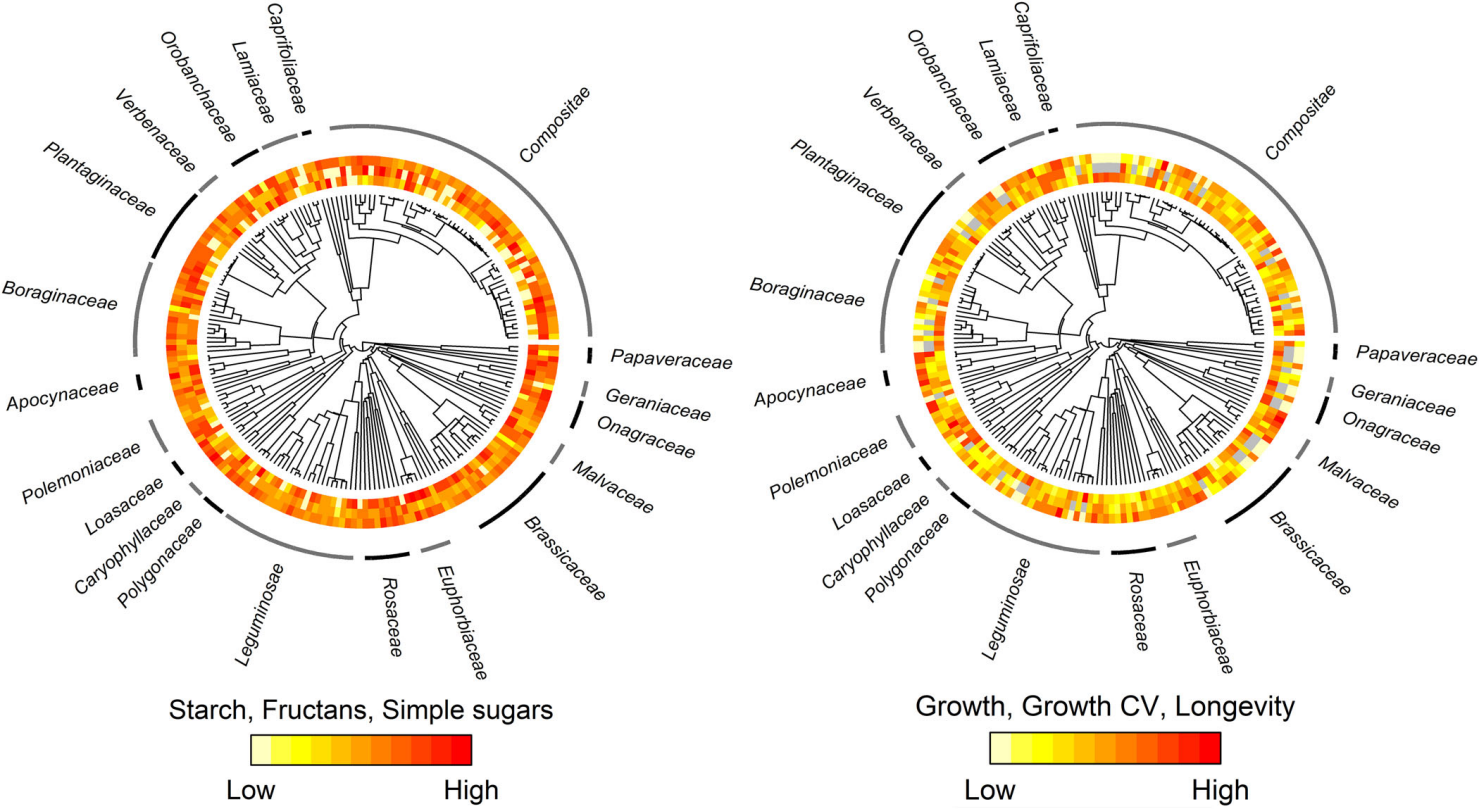
Phylogenetic tree of 201 studied species with visualisation of their relative trait values. Trait values are shown in colour, with lower values in light colours and higher values in reddish colours. The left panel shows colour‐coded content values for starch (inner circle – the circle closest to the tips of the phylogenetic tree), fructans (middle circle), and simple sugars (outer circle). The right panel shows colour‐coded values for mean growth rate (Growth), growth CV (coefficient of variation), and longevity. Missing values for the growth CV are indicated in grey. [Color figure can be viewed at wileyonlinelibrary.com]

We conducted the analyses using mean values of all variables for each species (across localities; when multiple individuals existed per locality, mean values were computed for that locality). This approach might have weakened the examined relationships for species with individuals sampled from multiple localities, particularly if significant intraspecific variability was present. Therefore, all analyses were also performed using data from randomly chosen localities for each species with representation in multiple localities, aiming to verify the robustness of the outcomes. In total, 201 species were included in all analyses, except for analyses concerning the CV of growth, where 154 species were considered due to some species having only one annual growth ring (Figure 2). Additionally, the analyses were separately carried out for the two herbaceous growth forms, which yielded a substantial number of observations (short‐lived herbs: 70 species; perennial herbaceous: 112 species). This separation allowed us to assess whether the influence of growth forms on these relationships was additive as assumed. We prepared the phylogenetic tree using the V. PhyloMaker package (version 0.1.0; Jin and Qian 2019) with scenario 2 which takes a mega‐tree (GBOTB. extended, based on Smith and Brown 2018) and randomly binds missing species below their genus crown node. We did all analyses in R (version 4.2.2; R Development Core Team 2022) using package caper (version 1.0.1; Orme et al. 2013) for phylogenetic linear models, package diversitree (version 0.9‐16; FitzJohn 2012) for phylogeny visualisation, and package smart for standardised major axis (version 3.4‐8; Warton et al. 2012).

## 3 Results

### 3.1 Relationships Between Studied Variables

**Figure 3:**
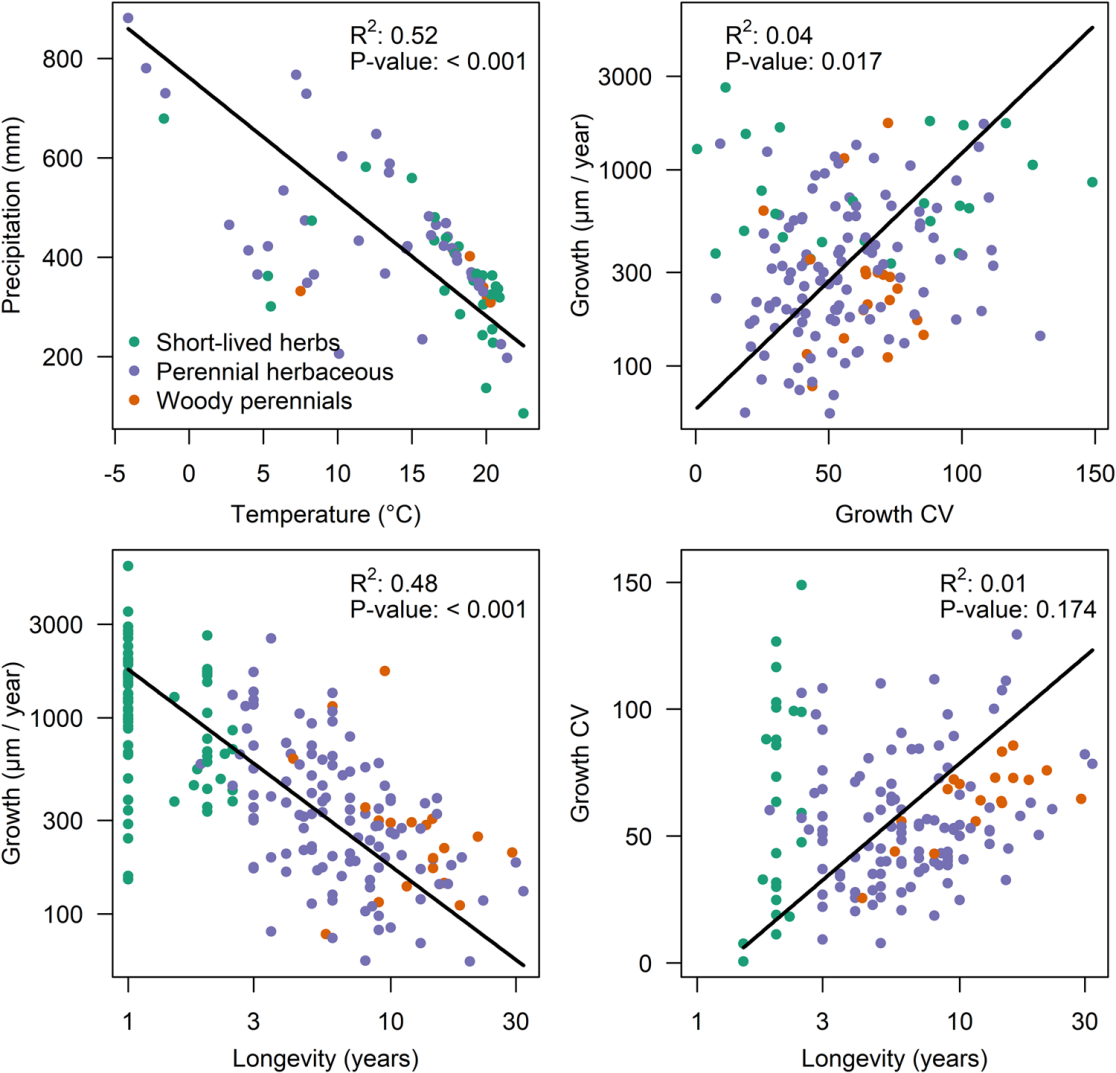
Relationships between environmental predictors (mean annual temperature and annual precipitation) and growth characteristics (mean ring width as a measure of radial growth rate and CV of radial growth) as well as plant age (number of growth rings in the oldest plant parts). The regression lines, derived from the standardised major axis (SMA), represent overall trends across all growth forms combined. Data points are colour‐coded by growth form (green: short‐lived herbs, purple: perennial herbaceous plants, orange: woody perennials) to highlight variability within and among these groups. [Color figure can be viewed at wileyonlinelibrary.com]

We found negative relationships between temperature and precipitation and between growth and longevity. There was a positive, yet weak, relationship between growth and its coefficient of variation and no relationship between growth CV and longevity (Figure 3). Among NSCs, we found only a weak positive relationship between the concentration of starch and simple sugars (Supporting Information Figure S1).

### 3.2 Effect of Growth Forms on Growth, Longevity and Carbohydrate Reserves

**Figure 4:**
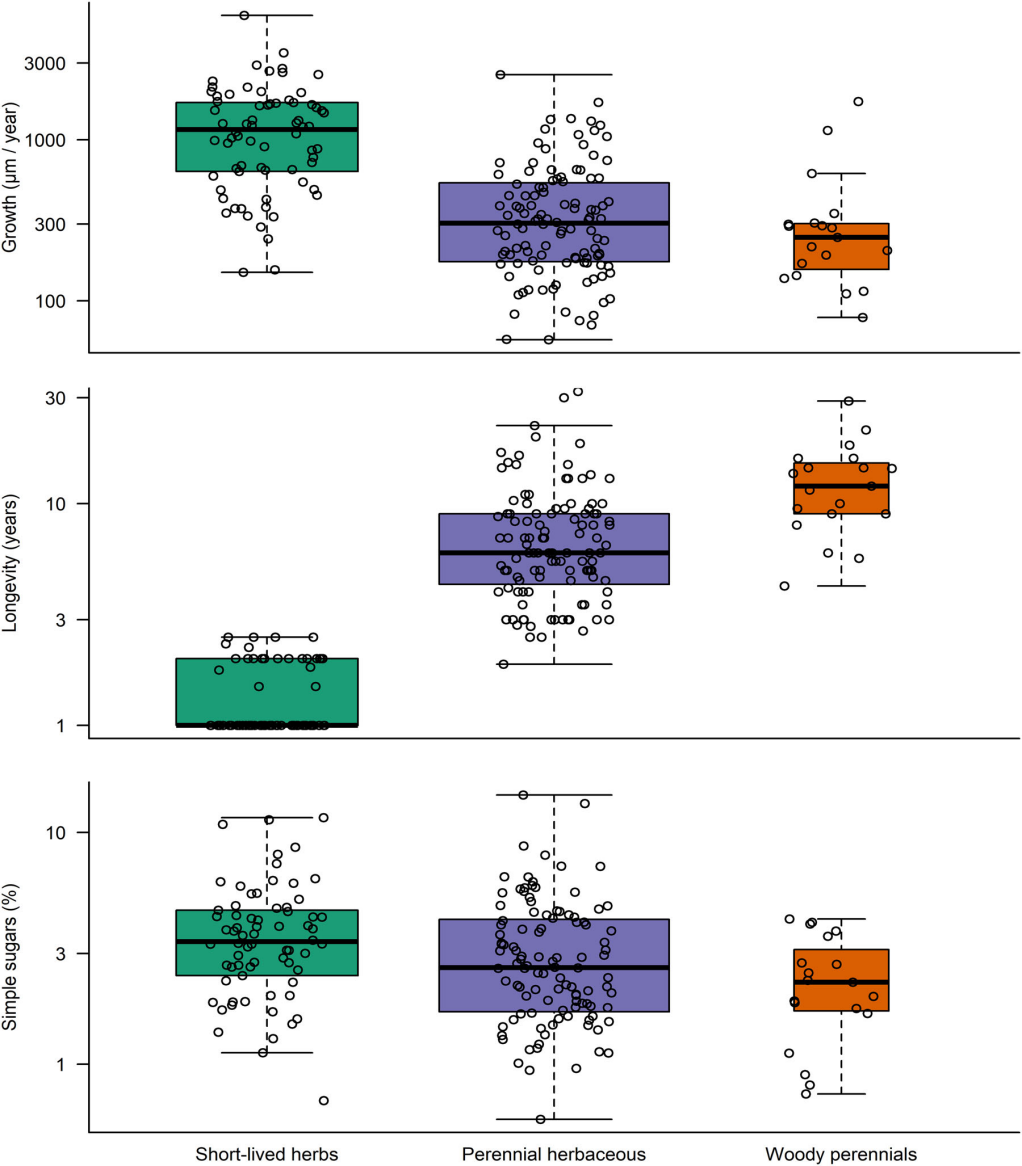
Growth, longevity and simple sugars for the three growth forms. The width of the boxes corresponds to the square root of the number of observations. The thick middle line denotes the median. Boxes range from the first to the third quartile, and whiskers range up to 3/2 of the interquartile range from the box. [Color figure can be viewed at wileyonlinelibrary.com]

We found a significant variation in mean annual growth, longevity and simple sugars among different plant growth forms (Supporting Information Table S3, Figure 4). On average, short‐lived herbs exhibited the highest growth rates compared to longer‐lived herbs and woody perennials. Among the investigated NSCs, only simple sugars exhibited significant variation between growth forms (Supporting Information Table S3), with the highest concentration in short‐lived plants and the lowest in woody perennials. While simple sugars were variable in the analysed samples, they never composed more than 15% of dry mass content.

### 3.3 Effect of Environmental Conditions on Carbohydrates, Radial Growth and Longevity

**Figure 5:**
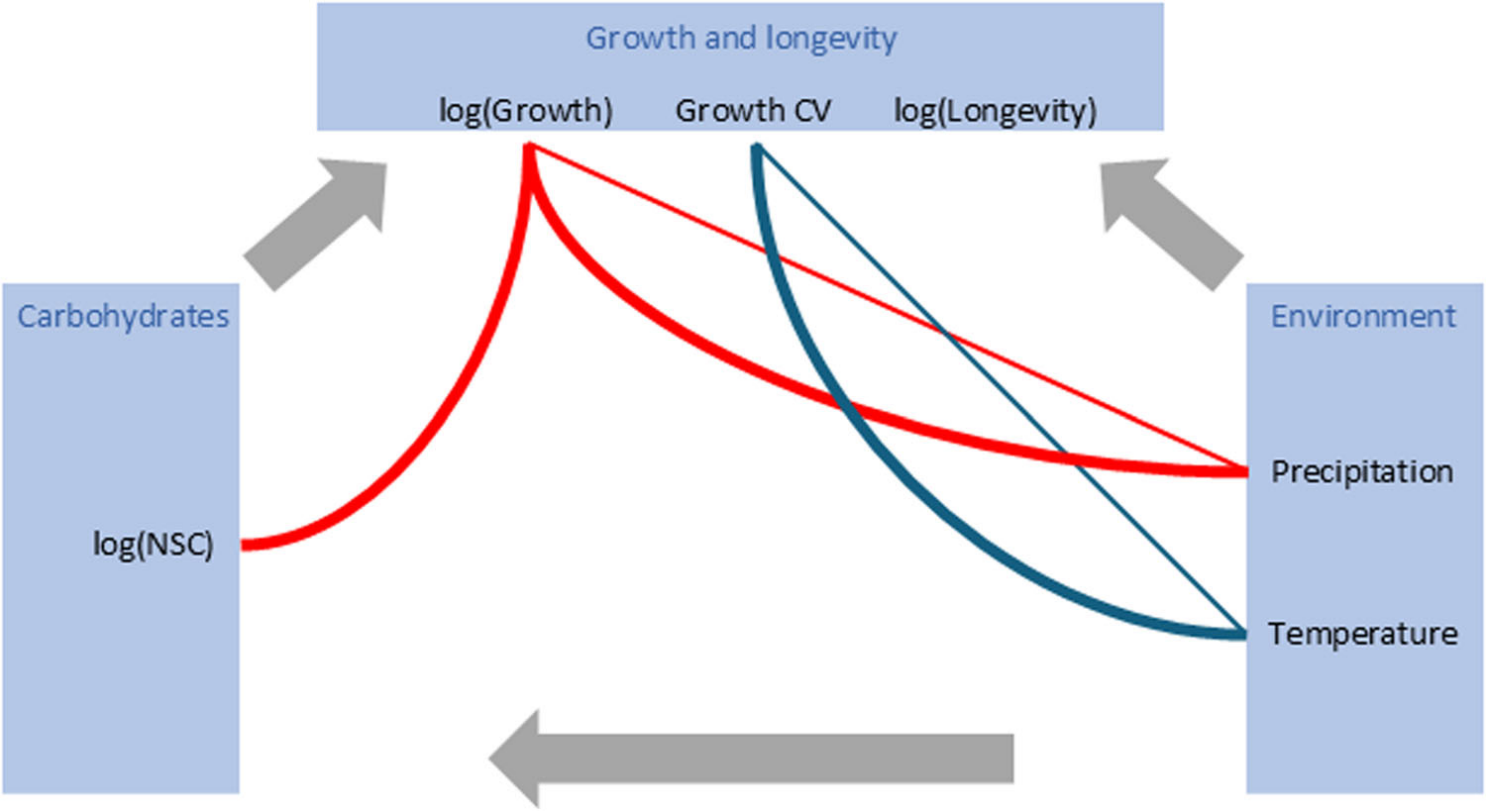
Diagram illustrating the complex interplay among environmental factors, total nonstructural carbohydrates (NSCs), and their influence on growth and longevity. Red lines denote negative effects, while blue lines positive effects (Supporting Information Table S3). Curved lines represent analyses accounting for both carbohydrate compounds and environmental variables. The “log” notation signifies natural logarithm, and “CV” represents the coefficient of variation. [Color figure can be viewed at wileyonlinelibrary.com]

We did not find any significant effect of the climatic variables on total NSCs. Environmental conditions affected starch and fructan content but explained only a small amount of variation, whereas no effect was found on simple sugars (Supporting Information Table S3). Effects of both precipitation and temperature on starch were positive, while fructans were negatively linked to precipitation (Figure 5). We found a direct effect of environmental conditions on growth and growth variability (Growth CV) but not longevity (Supporting Information Table S3). Precipitation was generally negatively related to growth, and temperature was positively related to CV of growth. Higher radial growth was identified in plants from drier conditions. In contrast, more variable growth was associated with higher temperatures (Figure 3).

### 3.4 Relationships Between Carbohydrates, Radial Growth Rates and Longevity

**Figure 6:**
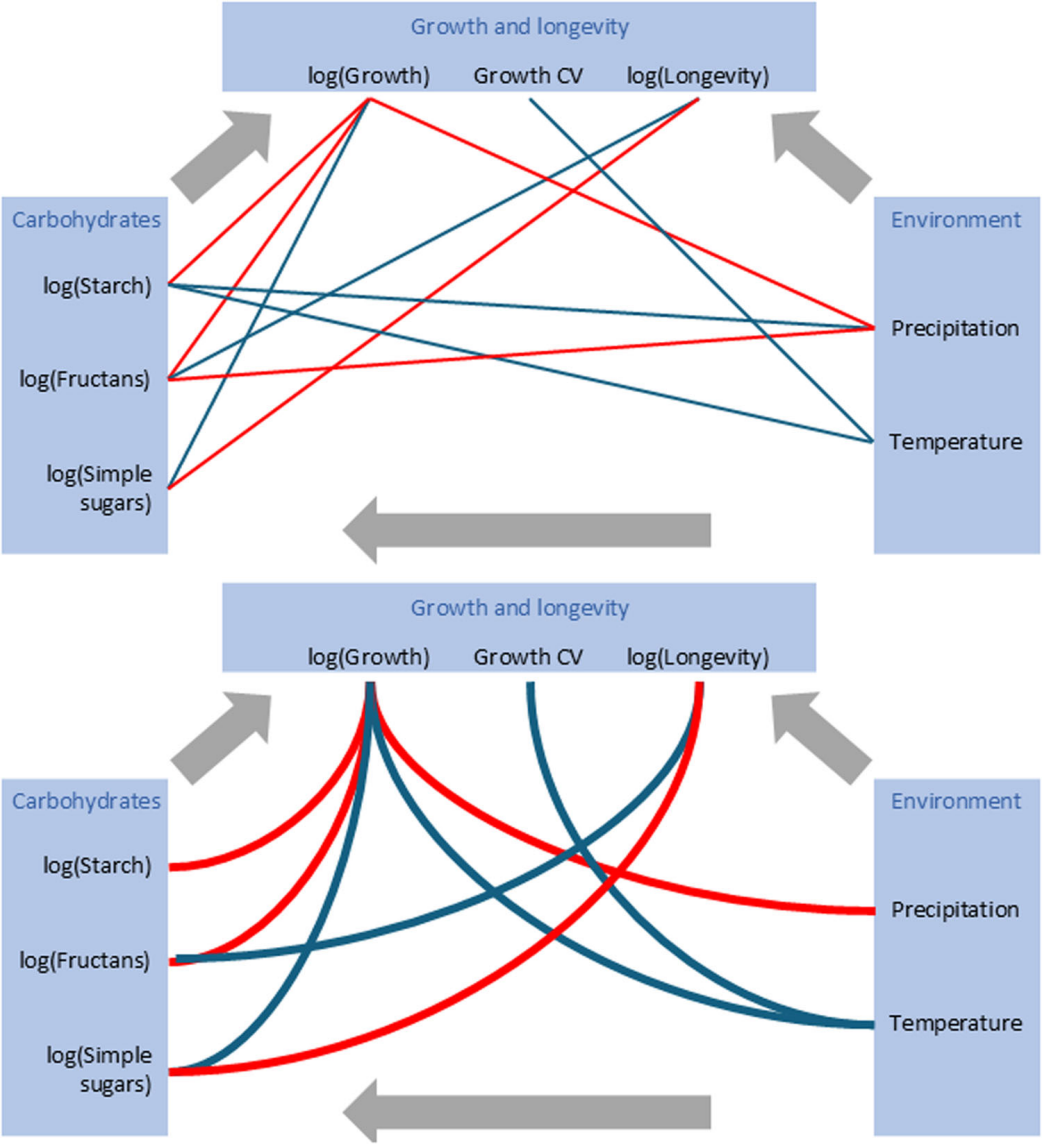
Diagrams illustrating the complex interplay among environmental factors, carbohydrate compounds, and their influence on growth and longevity. Red lines denote negative effects, while blue lines positive effects (Supporting Information Table S3). Curved lines represent analyses accounting for both carbohydrate compounds and environmental variables. The “log” notation signifies natural logarithm, and “CV” represents the coefficient of variation. [Color figure can be viewed at wileyonlinelibrary.com]

A negative relationship between total NSCs and growth was observed only after accounting for precipitation and temperature (Figure 5). All three types of analysed NSC groups had a significant relationship with growth (Supporting Information Table S3). We observed high mean radial growth in species with low starch and fructans but high levels of simple sugars. Additionally, higher fructan and simple sugar contents were significantly coupled with plants exhibiting higher longevity, while no NSCs showed any relationship with the variability of annual growth (CV of growth; Supporting Information Table S3). Fructans had a positive relationship with longevity, whereas simple sugars had a negative relationship (Figure 6). All these effects were found in both analyses with and without accounting for environmental variables.

### 3.5 Robustness Check and Phylogenetic Inertia

The estimated phylogenetic signal (λ) in individual analyses varied from very low (λ = 0) to moderate (λ = 0.44) (where λ = 0 corresponds to no phylogenetic signal and λ = 1 to the Brownian motion model of evolution), indicating a minor influence of phylogeny on these relationships (Supporting Information Table S3). Results showed little changes when utilising individuals from random localities instead of species averages: the direct effect of precipitation on growth was no longer significant (*p* = 0.074), the direct effect of temperature on NSCs became significant (*p* = 0.042), and the indirect effect of precipitation on growth was no longer significant (*p* = 0.061). When we analysed herbaceous growth forms separately, there was no change in the direction of identified relationships (Supporting Information Figure S2; many relationships identified for the data set were no longer significant for separate growth forms – which was expected since the number of species was lower).

## 4 Discussion

### 4.1 Trade‐Offs Between Growth, Longevity and Environmental Factors

The results show that increased temperature and lower precipitation positively impact plant growth and variability, which suggests that plants in drier or more thermally variable environments may adopt faster growth strategies (Dolezal et al. 2019). However, growth was inversely related to plant age, reflecting a trade‐off where plants with rapid growth may sacrifice longevity (Blumstein et al. 2022). This supports the growth‐longevity trade‐off hypothesis (Chondol et al. 2023), indicating that plants with limited resources (semi‐desert, cold alpine zones) may prioritise faster growth in challenging environments, likely due to selective pressures favoring quick resource utilisation (Arendt 1997; Black et al. 2008).

The weak relationship between NSCs and environmental factors, except for a modest effect of precipitation on starch and fructans, suggests that NSC accumulation is driven more by internal physiological requirements and growth strategies than by direct climatic influence. This finding challenges the carbon surplus hypothesis, which posits passive NSC accumulation due to climatic constraints and instead suggests that plants actively regulate their carbohydrate reserves according to growth and persistence needs (Wiley et al. 2013; Dietze et al. 2014).

### 4.2 Carbohydrate Trade‐Offs With Growth and Longevity

The debate over whether NSCs are actively accumulated to boost plant growth and longevity under carbon stress or passively build up due to growth limits is ongoing (Wiley et al. 2013; Dietze et al. 2014). Our findings align with studies indicating a trade‐off between storage and growth, suggesting plants actively allocate carbon to storage (Sala et al. 2012; Blumstein et al. 2022). We show the negative association between starch and growth, combined with the role of fructans in supporting longevity, suggesting that plants regulate NSC composition to manage energy and stress tolerance trade‐offs. Starch may serve as a stable energy reserve under constrained growth conditions, while fructans' role in resilience further supports longevity in more challenging habitats, such as deserts and alpine areas (Van den Ende and Oner 2023). Starch displays regulated behaviour, while simple sugars adapt flexibly to meet high metabolic demands, maximising growth in favourable conditions and helping plants manage water scarcity (Martínez‐Vilalta et al. 2016; Tixier et al. 2018; Aronson et al. 1992; Guo et al. 2020). Simple sugars are actively mobilised for growth, particularly in short‐lived species, supporting rapid development and reproduction in transient environments (Chlumská et al. 2022).

Simple sugars, found at higher levels in short‐lived herbs, support fast growth but may compromise longevity, as indicated by the negative association between simple sugars and persistence. Conversely, fructans are more prevalent in long‐lived species, such as semidesert woody shrubs and alpine perennial herbs, suggesting they play a role in stabilising cellular functions under stress and enhancing resilience (Rosas et al. 2013). Fructans contribute to longevity by stabilising cell hydration and aiding stress tolerance (Van den Ende 2013; Livingston et al. 2009; Suprasanna et al. 2016). This aligns with findings on fructans as multifunctional molecules aiding in stress tolerance, including drought resistance and highlights the differential role of NSC compounds in supporting distinct survival strategies depending on growth form. These varying NSC roles support the growth‐longevity trade‐off hypothesis, where different NSCs contribute to growth or longevity, balancing carbon use in plant life strategies (Smith and Stitt 2007; Sulpice et al. 2009; Huang et al. 2019; Blumstein et al. 2023).

### 4.3 NSCs and Radial Growth: Controlling for Confounding Factors

Our study revealed a significant negative relationship between total NSCs and radial growth in the sampled plant species after accounting for the environmental variations, life strategies and phylogenetic differences. The considerable variability in total NSC content makes it challenging to establish direct links between growth patterns and total NSC content in an interspecific context (Fung 2000; Huang et al. 2019). This result highlights that plant species' different carbon allocation strategies lead to a high variation in carbon utilisation. Therefore, one must consider variations in the carbon supply (source limitations, photosynthesis) when conducting interspecific studies exploring relationships that include total NSC content (Fung 2000; Sala et al. 2012; Blumstein et al. 2022). Previous studies have shown that for highly productive plant species, variation in carbon supply can blur the trade‐off between growth and carbon sinks (van Noordwijk and de Jong 1986; Osnas et al. 2018; Agrawal 2020). Indeed, a previous study found that when controlling for the variation in carbon supply, the relationship between growth and storage filled flipped from a positive association to a negative one (Blumstein et al. 2022). These findings underscore the importance of considering plant species' inherent environmental conditions, life strategies and phylogenetic differences when investigating the effect of total NSCs on interspecific variations in growth, growth variation and longevity among plant species.

### 4.4 Impacts of Individual NSC Compounds on Growth and Longevity

Our findings confirm that starch, fructans and simple sugars significantly influence annual growth rates and plant persistence. Specifically, simple sugars appear to be utilised from the NSC pool to boost growth, albeit at a cost to longevity. This aligns with other studies showing that plants modulate carbon fluxes within the simple sugars NSC pool to maintain steady growth under nonoptimal conditions, thereby avoiding carbon starvation (Gibon et al. 2009; Hartmann et al. 2013; Huang et al. 2019). The positive correlation observed between high annual radial growth and simple sugar levels in short‐lived desert species, like the sugar‐rich *Chylismia claviformis* and *Palafoxia arida* from the Sonoran Desert, likely arises from the greater availability of simple sugars for energy production and other metabolic processes (Couée et al. 2006; Guo et al. 2020; Blumstein et al. 2023). While starch undergoes degradation into glucose, especially under stress like drought or nutrient deficiency (Stitt and Zeeman 2012; Janeček et al. 2015), plants efficiently mobilise and utilise these sugars with fewer constraints during heightened metabolic demand to avoid carbon starvation (Smith and Stitt 2007; Sulpice et al. 2009). The negative correlation between starch and growth likely reflects its role as a tightly regulated reserve, accumulated at the cost of immediate growth and broken down strategically under stress (Chapin et al. 1990; Wiley et al. 2013; Martínez‐Vilalta et al. 2016). While previous studies also link starch and simple sugars to growth (Rosas et al. 2013; Piper et al. 2017), our study's expanded species range and controls for environmental, growth form and phylogenetic differences strengthen these findings. The phylogenetic influence was minimal, and the relationship between specific NSC compounds and growth and longevity was evident even without controlling for confounding factors. Robustness checks using individual rather than species‐averaged data showed only minor relationship changes.

The positive link between fructans and longevity likely stems from their multifunctionality beyond simple storage, with some plants substituting fructans for starch (Livingston et al. 2009; Pollock 1986; Hendry 2008; Van den Ende and Oner 2023). Fructans serve as reserve carbohydrates, membrane stabilisers, osmoprotectants and mediators of stress tolerance, enhancing plant fitness and resilience and likely supporting greater longevity (Livingston et al. 2009; Van den Ende 2013). Our findings underscore the multifunctionality of fructans, as higher fructan levels correlate with increased longevity in slow‐growing desert shrubs and alpine herbs and shrubs, while efficient starch breakdown into energy‐rich sugars promotes growth and reproduction in herbaceous species (Couée et al. 2006; Livingston et al. 2009; Hartmann and Trumbore 2016; Blumstein et al. 2023). These results highlight the distinct roles of NSCs in carbon allocation strategies, supporting the growth‐longevity trade‐off hypothesis, which suggests NSCs actively support growth and longevity rather than simply accumulating passively due to growth constraints (Prescott et al. 2020; Zepeda et al. 2022).

### 4.5 Phylogenetic Influence and Robustness

The low to moderate phylogenetic signal across analyses suggests limited evolutionary constraint on NSC‐related traits, indicating that adaptation to local environmental conditions plays a stronger role than phylogenetic history. The robustness of results across random localities and different analyses further supports the stability of the identified relationships, confirming that the observed patterns are broadly applicable across diverse taxa and environmental gradients. These findings highlight the importance of specific NSCs in mediating growth‐longevity trade‐offs, regardless of their evolutionary origins, with simple sugars favoring growth at the cost of persistence and fructans enhancing resilience and longevity. The active role of NSCs in adaptive strategies reflects a complex carbon allocation mechanism that optimises survival under varying environmental conditions. Future studies should investigate the mechanistic basis of NSC regulation across additional taxa and climates, emphasising long‐term observations to clarify further how environmental change influences these trade‐offs,

including recent climate warming and associated phenomena such as increased desertification in drylands and thermophilization of alpine regions.

## 5 Conclusions

Our study examined the trade‐offs between carbohydrate reserves, growth and longevity in over 200 vascular dicot plant species across diverse thermal and precipitation regimes in the Western United States. Our findings indicate that increased temperature and lower precipitation positively impact plant growth, suggesting that species in drier or more thermally variable environments may adopt faster growth strategies. However, we observed a trade‐off where rapid growth often correlates with reduced longevity, supporting the growth‐longevity trade‐off hypothesis. In resource‐limited environments, such as semideserts and cold alpine zones, plants may prioritise quicker growth due to selective pressures favoring efficient resource utilisation. Interestingly, our results show a weak relationship between nonstructural carbohydrates and environmental factors, challenging the carbon surplus hypothesis. Instead, we suggest that internal physiological needs and growth strategies primarily drive NSC accumulation. This research highlights the active role of NSCs in regulating energy and stress tolerance, revealing a complex interplay between different carbohydrate compounds. We found that while simple sugars are crucial for boosting growth, they may compromise longevity. Conversely, fructans are associated with resilience and stability in challenging habitats. This differentiation emphasises the necessity of considering individual NSC compounds when studying plant growth and longevity, particularly in light of climate change and its impact on environmental conditions. Our study also confirms the significant negative relationship between total NSCs and radial growth after accounting for various confounding factors. The considerable variability in NSC content underscores the importance of understanding carbon allocation strategies in an interspecific context. The low to moderate phylogenetic signal in our analyses indicates that local adaptations may play a more substantial role than evolutionary history. In conclusion, our research enhances the understanding of growth‐longevity trade‐offs and carbon allocation strategies in plants, emphasising the importance of specific NSCs in adapting to environmental conditions. Future studies should investigate the mechanistic basis of NSC regulation across various taxa and climates, particularly in the context of ongoing climate change.

## Conflicts of Interest

The authors declare no conflicts of interests.

## Supporting information

Supporting information.

## Data Availability

The data that support the findings of this study are available from the corresponding author upon reasonable request.
