# Supplementary material for: Trade‐Offs Between Growth, Longevity, and Storage Carbohydrates in Herbs and Shrubs: Evidence for Active Carbon Allocation Strategies
Source: Plant Cell Environ. 2025 Feb 27;48(6):4505–17. doi: 10.1111/pce.15444 (PMC12050394; doi:10.1111/pce.15444)
Supplement: Supplementary file 1 — Supporting information. [file PCE-48-4505-s001.docx]

**Trade-offs Between Growth, Longevity, and Storage Carbohydrates in Herbs and Shrubs: Evidence for Active Carbon Allocation Strategies**

**Table S1.** Summary information about various localities, including geographical, climatic, and elevation data. **Name of locality**: The specific location or area being referenced. **Elevation (m):** The height of the locality above sea level, measured in meters. **Latitude:** The geographical coordinate specifying the north-south position of the locality. **Longitude:** The geographical coordinate specifying the east-west position of the locality. **Annual Mean Temperature (ºC):** The average temperature of the locality over a year, measured in degrees Celsius. **Annual Precipitation (mm):** The total rainfall or precipitation received in a year, measured in millimeters. **Range of Elevation:** The lowest elevation is 533 m (Organ Pipe National Park). The highest elevation is 4313 m (Mt. Evans). **Temperature Variability:** The highest annual mean temperature is 22.5°C (Pinto Basin Road). The lowest annual mean temperature is -4.1°C (Mt. Evans). **Precipitation Patterns:** The driest location is Pinto Basin Road, which has only 86 mm of annual precipitation. The wettest location is Mt. Evans with 881 mm of annual precipitation.

| Name of locality | Elevation (m) | Latitude | Longitude | Annual Mean Temperature (ºC) | Annual Precipitation (mm) |
| --- | --- | --- | --- | --- | --- |
| Arivaca Lake | 1053 | 31.6090 | 111.3873 | 17.3 | 438 |
| Baboquivari | 1887 | 31.7872 | 111.6271 | 15 | 559 |
| Bajada wash trail | 775 | 32.2712 | 111.2082 | 19.6 | 352 |
| Barker Dam | 1299 | 34.0289 | 116.1464 | 14.9 | 311 |
| Capitol Reef NP | 1662 | 38.2082 | 111.1721 | 10.1 | 206 |
| Chiricahua Mts | 1415 | 32.0101 | 109.4398 | 14.7 | 422 |
| Chiricahua Mts, Up hill | 2446 | 31.9185 | 109.2702 | 10.3 | 603 |
| Coronado National Park | 852 | 32.2509 | 110.6653 | 17.8 | 412 |
| Craters of the Moon NM | 1753 | 43.4458 | 113.5343 | 5.3 | 362 |
| Dinosaurus National Park | 2348 | 40.4546 | 109.0154 | 4.6 | 365 |
| Fort Collins | 1618 | 40.8131 | 104.7131 | 8.4 | 365 |
| Fort Hays | 609 | 38.8442 | 99.3167 | 11.9 | 582 |
| Fort Keogh | 815 | 46.4050 | 105.9498 | 7.5 | 332 |
| Fossil Creek | 1106 | 34.4107 | 111.6068 | 13.5 | 588 |
| Great Sand Dunes | 2482 | 37.7285 | 105.5171 | 5.5 | 301 |
| Joshua Tree National Park | 658 | 33.6828 | 115.7989 | 20 | 137 |
| Kerr Jar Trail | 790 | 31.7130 | 110.8746 | 19.7 | 340 |
| Madera Canyon | 1508 | 39.6011 | 105.6347 | 12.6 | 648 |
| Mt. Evans | 4313 | 32.6589 | 109.8722 | -4.1 | 881 |
| Mt. Graham | 3020 | 31.7131 | 110.8747 | 7.2 | 767 |
| Mt. Lemmon | 2310 | 32.3380 | 110.6994 | 12.6 | 648 |
| Mt. Lemmon - road | 1380 | 44.8132 | 110.4385 | 14.4 | 558 |
| Mt. Washburn | 2818 | 32.2084 | 111.1296 | -1.7 | 679 |
| North Tucson | 769 | 32.1730 | 112.2092 | 19.8 | 305 |
| Organ Pipe National Park | 533 | 31.9439 | 112.8164 | 21 | 225 |
| Patagonia lake | 1170 | 31.4940 | 110.8579 | 16.3 | 444 |
| Picacho Peak | 987 | 32.6356 | 111.4012 | 20.8 | 336 |
| Picture Rocks, Golden Gate Road | 704 | 32.3155 | 111.1565 | 19.9 | 339 |
| Pima Canyon | 939 | 32.3573 | 110.9346 | 18.9 | 402 |
| Pinto Basin Road | 539 | 33.1748 | 115.8893 | 22.5 | 86 |
| Piper Spring Road | 658 | 32.9755 | 110.7523 | 20.4 | 363 |
| Picacho Peak slopes | 598 | 32.6467 | 111.4278 | 20.9 | 319 |
| Richard Genser | 822 | 32.2017 | 111.0451 | 19.8 | 331 |
| Rockies NP | 3239 | 40.4127 | 105.8172 | -1.6 | 730 |
| Saguaro East | 871 | 32.2035 | 110.7114 | 19.5 | 349 |
| San Pedro River | 1163 | 31.7248 | 110.1936 | 17.2 | 333 |
| Santa Rita Experimental Range | 1200 | 31.8331 | 110.8523 | 17.4 | 441 |
| Sunset Crater | 2092 | 35.3467 | 111.5099 | 8.6 | 425 |
| Sycamore Canyon | 1114 | 34.9012 | 112.0667 | 7.8 | 474 |
| Tanque Verde | 855 | 31.4132 | 110.9075 | 14.6 | 417 |
| Tumamoc Hill | 766 | 32.2249 | 111.0080 | 16.5 | 434 |
| Ventana canyon | 964 | 32.3334 | 110.8549 | 20.3 | 309 |
| White Sand Dunes New Mexico | 1210 | 32.8188 | 106.2656 | 15.7 | 235 |
| Wyoming Jackson | 1908 | 43.5139 | 110.7512 | 2.7 | 465 |
| Yetman trail | 861 | 32.2071 | 111.0688 | 19.8 | 331 |

**Table S2.** Detailed information about various plant species collected in the Western United States and used for this study is categorized into columns. **Species**: Scientific name of the plant. **Family**: The botanical family to which the plant belongs. **Growth Form**: Describes the plant's life habits (e.g., short-lived herbs, perennial herbaceous, woody perennials). **Organ**: The primary organ or part of the plant associated with its longevity or growth (e.g., root, root collar, rhizome, stem base). **Max Age**: The species' maximum recorded age (in years). **Localities**: Specific regions or locations where the species has been collected. Plant persistence (**Longevity)**: Short-lived herbs typically have a max age of 1-2 years. For example, *Amsinckia menziesii* lives for 1 year. Perennial herbs like *Calliandra eriophylla* and *Salvia arizonica* exhibit extended lifespans, up to 29 years. Woody perennials like shrubby Ambrosia cordifolia and Amorpha canescens reach ages 26 and 29 years, respectively. **Geographic Spread**: Localities range widely across natural parks, trails, canyons, and experimental ranges (e.g., Joshua Tree National Park, Mt. Graham, Northern Great Plains). The **root collar** is the part of a plant where the roots and the stem meet, typically located at or near the soil surface. It serves as the transition zone between the underground root system and the plant's above-ground parts. The **stem base** is the lower portion of a plant's stem, situated just above the root collar and close to the ground. A **rhizome** is a modified, underground stem that grows horizontally beneath the soil surface or just above it. Unlike roots, rhizomes have nodes, internodes, and buds, which can give rise to new shoots and roots. They are significant in vegetative reproduction, food storage, and plant survival.

| Species | Family | Growth Form | Organ | Max Age | Localities |
| --- | --- | --- | --- | --- | --- |
| Abronia villosa | Nyctaginaceae | Perennial herbaceous | root | 4 | Pinto Basin Road |
| Abutilon abutiloides | Nyctaginaceae | Perennial herbaceous | root collar | 18 | Ventana Canyon, Pima Canyon |
| Achillea millefolium var. borealis | Asteraceae | Short-lived herbs | root | 1 | Fort Hays |
| Acmispon neomexicanus | Fabaceae | Perennial herbaceous | root | 3 | Chiricahua Mts |
| Acmispon rigidus | Fabaceae | Perennial herbaceous | root | 8 | Fossil Creek, Barker Dam |
| Acmispon strigosus | Fabaceae | Short-lived herbs | root | 1 | Joshua tree National Park |
| Acourtia wrightii | Asteraceae | Perennial herbaceous | root collar | 11 | Yetman trail |
| Agastache breviflora | Lamiaceae | Perennial herbaceous | root | 3 | Mt. Graham |
| Agrimonia striata | Rosaceae | Perennial herbaceous | root collar | 3 | Mt. Lemmon |
| Ambrosia cordifolia | Asteraceae | Woody perennials | stem base | 26 | Pima Canyon, Yetman trail |
| Ambrosia deltoidea | Asteraceae | Woody perennials | stem base | 17 | Tumamoc Hill, Yetman Trail, Kerr Jar Trail |
| Ambrosia psilostachya | Asteraceae | Short-lived herbs | root | 1 | Fort Hays |
| Amorpha canescens | Fabaceae | Woody perennials | stem base | 29 | Northern Great Plains |
| Amsinckia menziesii | Boraginaceae | Short-lived herbs | root | 1 | Santa Rita Experimental Range, Pima Canyon |
| Amsinckia menziesii var. intermedia | Boraginaceae | Short-lived herbs | root | 2 | Pima Canyon |
| Anagallis arvensis | Primulaceae | Short-lived herbs | root | 1 | Tanque Verde |
| Anisacanthus thurberi | Acanthaceae | Woody perennials | stem base | 10 | Baboquivari |
| Antennaria marginata | Asteraceae | Perennial herbaceous | root collar | 9 | Madera Canyon |
| Argemone pleiacantha | Papaveraceae | Short-lived herbs | root | 1 | Baboquivari, Kerr Jar Trail |
| Arnica latifolia | Asteraceae | Perennial herbaceous | root collar | 8 | Mt. Washburn |
| Artemisia dracunculus | Asteraceae | Perennial herbaceous | root | 7 | Northern Great Plains |
| Artemisia frigida | Asteraceae | Woody perennials | stem base | 17 | Northern Great Plains |
| Artemisia scopulorum | Asteraceae | Woody perennials | stem base | 7 | Mt. Evans |
| Artemisia tridentata | Asteraceae | Woody perennials | stem base | 9 | Fort Keogh |
| Asclepias asperula | Apocynaceae | Perennial herbaceous | root collar | 10 | Patagonia lake |
| Asclepias viridis | Apocynaceae | Perennial herbaceous | root collar | 15 | Fort Hays |
| Astragalus gracilis | Fabaceae | Perennial herbaceous | root collar | 10 | Fort Collins |
| Astragalus mollissimus | Fabaceae | Perennial herbaceous | root collar | 3 | Fort Hays |
| Brickellia coulteri | Asteraceae | Woody perennials | stem base | 19 | Picture Rocks blizko Golden Gate Road, Pima Canyon |
| Calliandra eriophylla | Fabaceae | Woody perennials | rhizome | 23 | Baboquivari, Coronado National Park, Tanque verde |
| Callirhoe involucrata | Malvaceae | Perennial herbaceous | root collar | 9 | Fort Hays |
| Carphochaete bigelovii | Asteraceae | Perennial herbaceous | rhizome | 8 | Baboquivari |
| Castilleja applegatei | Orobanchaceae | Perennial herbaceous | root | 6 | Sycamore Canyon |
| Castilleja exserta | Scrophulariaceae | Short-lived herbs | root | 1 | North Tucson |
| Castilleja integra | Orobanchaceae | Perennial herbaceous | root collar | 13 | Chiricahua Mts |
| Castilleja pallescens | Orobanchaceae | Perennial herbaceous | root | 6 | Mt. Washburn |
| Chaenactis douglasii | Asteraceae | Perennial herbaceous | root collar | 5 | Craters of the Moon |
| Chaenactis fremontii | Asteraceae | Short-lived herbs | root | 1 | Joshua Tree National Park |
| Chorizanthe brevicornu | Polygonaceae | Short-lived herbs | root | 1 | Picacho Peak slopes, Piper Spring Road |
| Chylismia claviformis | Onagraceae | Short-lived herbs | root | 1 | Joshua Tree National Park |
| Chylismia claviformis subsp. peirsonii | Onagraceae | Short-lived herbs | root | 1 | Pinto Basin Road |
| Cleomella serrulata | Cleomaceae | Short-lived herbs | root | 1 | Great Sand dunes |
| Conyza canadensis | Asteraceae | Short-lived herbs | root | 1 | Fort Hays |
| Crossosoma bigelovii | Crossosomataceae | Woody perennials | stem base | 16 | Pima Canyon |
| Cryptantha micrantha | Boraginaceae | Short-lived herbs | root | 1 | Santa Rita Experimental Range |
| Cryptantha pterocarya | Boraginaceae | Short-lived herbs | root | 1 | Bajada wash trail |
| Cymopterus lemmonii | Apiaceae | Perennial herbaceous | root | 5 | Mt. Graham |
| Dalea pringlei | Fabaceae | Perennial herbaceous | root collar | 9 | Tanque Verde |
| Dalea purpurea | Fabaceae | Woody perennials | stem base | 9 | Northern Great Plains |
| Delphinium scaposum | Ranunculaceae | Short-lived herbs | root | 1 | Baboquivari |
| Descurainia pinnata | Brassicaceae | Short-lived herbs | root | 1 | Pima Canyon |
| Dieteria canescens | Asteracceae | Short-lived herbs | root | 2 | Wyoming Jackson |
| Draba grayana | Brassicaceae | Perennial herbaceous | root | 9 | Mt. Evans |
| Draba incerta | Brassicaceae | Perennial herbaceous | root collar | 18 | Mt. Washburn |
| Drymocallis glandulosa | Rosaceae | Perennial herbaceous | root | 11 | Craters of the Moon |
| Dryopetalon runcinatum | Brassicaceae | Short-lived herbs | root | 3 | Picacho Peak |
| Emmenanthe penduliflora | Boraginaceae | Short-lived herbs | root | 1 | Joshua Tree National Park |
| Ephedra torreyana | Ephedraceae | Woody perennials | stem base | 24 | White Sand Dunes New Mexico |
| Ephedra trifurca | Ephedraceae | Woody perennials | stem base | 25 | Tumamoc Hill, Santa Rita Experimental Range |
| Eremothera boothii | Onagraceae | Short-lived herbs | root | 1 | Joshua Tree National Park |
| Eriastrum eremicum | Polemoniaceae | Short-lived herbs | root | 1 | Piper Spring Road |
| Ericameria nauseosa | Asteraceae | Woody perennials | stem base | 6 | White Sand Dunes New Mexico |
| Erigeron divergens | Asteraceae | Short-lived herbs | root | 1 | Tanque Verde |
| Erigeron oreophilus | Asteraceae | Perennial herbaceous | root collar | 5 | Mt. Graham |
| Erigeron simplex | Asteraceae | Perennial herbaceous | root collar | 6 | Mt. Evans |
| Eriogonum ovalifolium var. focarium | Polygonaceae | Perennial herbaceous | root | 4 | Craters of the Moon |
| Eriogonum umbellatum | Polygonaceae | Perennial herbaceous | root collar | 10 | Wyoming Jackson, Craters of the Moon |
| Eritrichium argenteum | Boraginaceae | Perennial herbaceous | root collar | 8 | Mt. Evans |
| Erodium cicutarium | Geraniaceae | Short-lived herbs | root | 3 | Pod Picacho Peak, Tumamoc Hill, Joshua Tree National Park |
| Erodium texanum | Geraniaceae | Short-lived herbs | root | 1 | Pod Picacho Peak, Tumamoc Hill, Picture Rocks Golden Gate Road |
| Erysimum capitatum | Brassicaceae | Perennial herbaceous | root collar | 3 | Mt. Graham |
| Erythranthe guttata | Phrymaceae | Short-lived herbs | root | 1 | Baboquivari |
| Eschscholzia californica | Papaveraceae | Short-lived herbs | root | 1 | Pod Picacho Peak, Joshua Tree National Park |
| Eschscholzia californica subsp. mexicana | Papaveraceae | Short-lived herbs | root | 1 | Yetman trail |
| Eulobus californicus | Onagraceae | Short-lived herbs | root | 1 | Pima Canyon |
| Euphorbia arizonica | Euphorbiaceae | Perennial herbaceous | root | 18 | Baboquivari |
| Euphorbia esula | Euphorbiaceae | Perennial herbaceous | root | 15 | Northern Great Plains |
| Euphorbia hyssopifolia | Euphorbiaceae | Perennial herbaceous | root collar | 10 | Picacho Peak slopes |
| Euphorbia pediculifera | Euphorbiaceae | Short-lived herbs | root | 2 | Piper Spring Road |
| Euphorbia polycarpa | Euphorbiaceae | Perennial herbaceous | root collar | 6 | Bajada wash trail |
| Evolvulus arizonicus | Convolvulaceae | Perennial herbaceous | root collar | 11 | Tanque Verde, Pima Canyon |
| Fallugia paradoxa | Rosaceae | Perennial herbaceous | root collar | 6 | Sunset Crater |
| Fouquieria splendens | Fouquieriaceae | Woody perennials | stem base | 18 | Kerr Jar Trail |
| Fragaria virginiana subsp. glauca | Rosaceae | Perennial herbaceous | root collar | 3 | Mt. Lemmon |
| Funastrum cynanchoides | Apocynaceae | Perennial herbaceous | root | 5 | Tanque Verde |
| Gaillardia spathulata | Asteraceae | Perennial herbaceous | root collar | 3 | Capitol Reef NP |
| Galium stellatum | Rubiaceae | Perennial herbaceous | root | 17 | Picacho Peak |
| Galium wrightii | Rubiaceae | Perennial herbaceous | root | 18 | Mt. Graham |
| Geranium caespitosum var. fremontii | Rosaceae | Perennial herbaceous | root collar | 4 | Mt. Evans |
| Geum rossii | Verbenaceae | Perennial herbaceous | root collar | 6 | Mt. Lemmon - road, Baboquivari, North Tucson, Arivaca Lake |
| Glandularia gooddingii | Asteraceae | Perennial herbaceous | root collar | 8 | Dinosaurs, Fort Hays |
| Grindelia squarrosa | Asteraceae | Short-lived herbs | root | 3 | Sycamore Canyon, Great Sand Dunes, Baboquivari |
| Gutierrezia sarothrae | Lamiaceae | Perennial herbaceous | root | 9 | Mt. Graham, Fort Keogh |
| Hedeoma hyssopifolia | Fabaceae | Perennial herbaceous | root collar | 16 | Wyoming Jackson |
| Hedysarum occidentale | Asteraceae | Short-lived herbs | root | 1 | Fort Hays |
| Helianthus annuus | Pteridaceae | Short-lived herbs | root | 1 | Baboquivari |
| Hemionitis fendleri | Pteridaceae | Perennial herbaceous | root | 7 | Baboquivari |
| Hemionitis wrightiana | Asteraceae | Short-lived herbs | root | 1 | Fort Hays |
| Heterotheca villosa | Asteraceae | Woody perennials | stem base | 8 | Mt. Evans, Fort Keogh, Fort Collins |
| Hymenoxys grandiflora | Asteraceae | Perennial herbaceous | root collar | 6 | Mt. Graham |
| Hymenoxys hoopesii | Polemoniaceae | Perennial herbaceous | rhizome | 5 | Mt. Graham, Great Sand Dunes |
| Ipomopsis aggregata | Asteraceae | Perennial herbaceous | root collar | 3 | Santa Rita Experimental Range |
| Isocoma tenuisecta | Malpighiaceae | Perennial herbaceous | root collar | 13 | Tanque Verde |
| Janusia gracilis | Boraginaceae | Perennial herbaceous | root collar | 9 | Picacho Peak slopes, Joshua Tree National Park |
| Johnstonella angustifolia | Krameriaceae | Short-lived herbs | root | 3 | Tumamoc Hill, Picture Rocks Golden Gate Road, Kerr Jar Trail |
| Krameria bicolor | Asteraceae | Woody perennials | stem base | 12 | Fort Hays |
| Lactuca serriola | Zygophyllaceae | Short-lived herbs | root | 1 | Tumamoc Hill, Picture Rocks Golden Gate Road, Kerr Jar Trail |
| Larrea tridentata | Brassicaceae | Woody perennials | stem base | 15 | Baboquivari |
| Lepidium densiflorum | Brassicaceae | Perennial herbaceous | root | 8 | Arivaca Lake |
| Lepidium lasiocarpum | Brassicaceae | Short-lived herbs | root | 1 | Picacho Peak slopes |
| Lepidium oblongum | Brassicaceae | Short-lived herbs | root | 1 | Pima Canyon, Yetman trail |
| Lepidium virginicum | Linaceae | Short-lived herbs | root | 1 | Mt. Graham |
| Linum lewisii | Boraginaceae | Perennial herbaceous | root collar | 8 | Northern Great Plains |
| Lithospermum occidentale | Fabaceae | Perennial herbaceous | root collar | 6 | Mt. Washburn |
| Lupinus caudatus | Fabaceae | Perennial herbaceous | root collar | 12 | Bajada Wash Trail, Santa Rita Experimental Range, Madera Canyon |
| Lupinus concinnus | Fabaceae | Short-lived herbs | root | 1 | Chiricahua Mts, Up hill |
| Lupinus neomexicanus | Fabaceae | Perennial herbaceous | root collar | 5 | Dinosaurs |
| Lupinus sericeus | Fabaceae | Perennial herbaceous | root collar | 4 | Joshua Tree National Park |
| Lupinus succulentus | Solanaceae | Short-lived herbs | root | 1 | Yetman trail |
| Lycium andersonii | Asteraceae | Woody perennials | stem base | 22 | Fort Hays |
| Machaeranthera tanacetifolia | Asteraceae | Perennial herbaceous | root collar | 9 | Tanque Verde, Joshua Tree National Park, Fort Collins |
| Malacothrix glabrata | Fabaceae | Short-lived herbs | root | 1 | Bajada wash trail |
| Marina parryi | Asteraceae | Perennial herbaceous | root collar | 10 | Sycamore Canyon |
| Melampodium leucanthum | Loasaceae | Perennial herbaceous | root collar | 13 | Baboquivari |
| Mentzelia albicaulis | Loasaceae | Short-lived herbs | root | 3 | Piper Spring Road |
| Mentzelia integra | Loasaceae | Short-lived herbs | root | 2 | White Sand Dunes New Mexico |
| Mentzelia procera | Asteraceae | Short-lived herbs | root | 2 | Tanque Verde |
| Microseris lindleyi | Lamiaceae | Short-lived herbs | root | 1 | Mt. Graham |
| Monarda citriodora subsp. austromontana | Boraginaceae | Perennial herbaceous | root collar | 5 | San Pedro River |
| Nama demissa | Solanaceae | Short-lived herbs | root | 1 | Tumamoc Hill, Bajada wash trail, Coronado National Park |
| Nicotiana obtusifolia | Brassicaceae | Perennial herbaceous | root collar | 7 | Chiricahua Mts, Up hill |
| Noccaea fendleri | Plantaginaceae | Perennial herbaceous | root | 6 | Pima Canyon |
| Nuttallanthus texanus | Onagraceae | Short-lived herbs | root | 1 | White Sand Dunes New Mexico |
| Oenothera hartwegii subsp. fendleri | Onagraceae | Perennial herbaceous | root collar | 11 | Richard Genser, Pima Canyon |
| Oxytropis sericea | Fabaceae | Perennial herbaceous | root collar | 5 | Mt. Washburn |
| Packera cana | Asteraceae | Short-lived herbs | root | 1 | Mt. Washburn |
| Packera neomexicana | Asteraceae | Short-lived herbs | root | 2 | Madera Canyon |
| Palafoxia arida | Asteraceae | Short-lived herbs | root | 1 | Pinto Basin Road |
| Pectocarya recurvata | Boraginaceae | Short-lived herbs | root | 3 | Picacho Peak slopes, Yetman trail |
| Pedicularis procera | Orobanchaceae | Perennial herbaceous | root collar | 10 | Mt. Graham |
| Pediomelum argophyllum | Fabaceae | Perennial herbaceous | root collar | 12 | Northern Great Plains |
| Penstemon albidus | Plantaginaceae | Perennial herbaceous | root collar | 11 | Fort Keogh, Fort Collins |
| Penstemon angustifolius | Plantaginaceae | Perennial herbaceous | root collar | 7 | Fort Collins |
| Penstemon barbatus | Plantaginaceae | Perennial herbaceous | root collar | 6 | Mt. Graham |
| Penstemon cyaneus | Plantaginaceae | Perennial herbaceous | root collar | 3 | Wyoming Jackson |
| Penstemon deaveri | Plantaginaceae | Perennial herbaceous | root collar | 5 | Mt. Graham |
| Penstemon palmeri | Plantaginaceae | Perennial herbaceous | root collar | 8 | Capitol Reef NP |
| Penstemon parryi | Plantaginaceae | Perennial herbaceous | rhizome | 7 | Mt. Lemmon - road, Baboquivari |
| Penstemon procerus | Plantaginaceae | Perennial herbaceous | root collar | 14 | Mt. Washburn |
| Perityle emoryi | Asteraceae | Perennial herbaceous | root collar | 5 | Picacho Peak |
| Perityle lemmonii | Asteraceae | Short-lived herbs | root | 2 | Pima Canyon |
| Phacelia campanularia | Boraginaceae | Short-lived herbs | root | 1 | Joshua Tree National Park |
| Phacelia crenulata | Boraginaceae | Short-lived herbs | root | 2 | Picacho Peak slopes |
| Phacelia cryptantha | Boraginaceae | Short-lived herbs | root | 3 | Craters of the Moon |
| Phacelia distans | Boraginaceae | Short-lived herbs | root | 1 | Pima Canyon |
| Phacelia sericea | Boraginaceae | Perennial herbaceous | root collar | 6 | Mt. Washburn, Mt. Evans |
| Phlox multiflora | Polemoniaceae | Perennial herbaceous | root collar | 20 | Mt. Washburn |
| Phlox tenuifolia | Polemoniaceae | Perennial herbaceous | root | 25 | Pima Canyon |
| Physaria gordonii | Brassicaceae | Perennial herbaceous | root collar | 5 | Picture Rocks Golden Gate Road, Yetman trail, Arivaca Lake |
| Picradeniopsis absinthifolia | Asteraceae | Perennial herbaceous | root collar | 6 | Tanque Verde, Coronado National Park |
| Plantago ovata | Plantaginaceae | Perennial herbaceous | root collar | 4 | Tumamoc Hill, Piper Spring Road, Pinto Basin Road |
| Polemonium pulcherrimum | Polemoniaceae | Perennial herbaceous | root collar | 5 | Mt. Washburn |
| Polemonium viscosum | Polemoniaceae | Perennial herbaceous | root collar | 6 | Rockies NP |
| Polygala alba | Polygalaceae | Perennial herbaceous | root | 7 | Northern Great Plains |
| Porophyllum gracile | Asteraceae | Perennial herbaceous | root collar | 19 | Bajada wash trail, Tanque Verde |
| Potentilla diversifolia | Rosaceae | Perennial herbaceous | root collar | 4 | Mt. Washburn |
| Rafinesquia neomexicana | Asteraceae | Short-lived herbs | root | 1 | Baboquivari |
| Rosa arkansana | Rosaceae | Woody perennials | stem base | 19 | Northern Great Plains |
| Rudbeckia laciniata | Asteraceae | Perennial herbaceous | root collar | 4 | Mt. Graham |
| Rumex hymenosepalus | Polygonaceae | Short-lived herbs | root | 2 | Baboquivari |
| Sabulina rubella | Caryophyllaceae | Perennial herbaceous | root collar | 10 | Mt. Evans |
| Salvia arizonica | Lamiaceae | Perennial herbaceous | root collar | 29 | Mt. Graham |
| Salvia columbariae | Lamiaceae | Short-lived herbs | root | 3 | Saguaro East, Joshua Tree National Park |
| Senecio atratus | Asteraceae | Perennial herbaceous | root collar | 5 | Rockies NP |
| Senecio bigelovii | Asteraceae | Short-lived herbs | root | 1 | Mt. Graham |
| Senecio flaccidus | Asteraceae | Perennial herbaceous | root collar | 4 | Baboquivari, Santa Rita Experimental Range |
| Senecio integerrimus | Asteraceae | Short-lived herbs | root | 2 | Mt. Washburn |
| Senna covesii | Fabaceae | Perennial herbaceous | root collar | 11 | Tumamoc Hill, Bajada wash trail, Baboquivari |
| Silene laciniata subsp. greggii | Caryophyllaceae | Perennial herbaceous | root collar | 6 | Mt. Graham |
| Silene scouleri | Caryophyllaceae | Perennial herbaceous | root collar | 4 | Mt. Graham |
| Sisymbrium irio | Brassicaceae | Short-lived herbs | root | 1 | Bajada Wash Trail, Santa Rita Experimental Range, Picacho Peak, Pima Canyon |
| Solidago rigida | Asteraceae | Perennial herbaceous | root collar | 3 | Northern Great Plains |
| Sonchus oleraceus | Asteraceae | Short-lived herbs | root | 2 | Picacho Peak |
| Sphaeralcea coccinea | Malvaceae | Perennial herbaceous | root collar | 9 | Pima Canyon, Fort Keogh |
| Sphaeralcea laxa | Malvaceae | Perennial herbaceous | root collar | 19 | Richard Genser, Yetman trail |
| Stachys coccinea | Lamiaceae | Perennial herbaceous | root collar | 10 | Patagonia lake |
| Streptanthus carinatus | Brassicaceae | Short-lived herbs | root | 1 | Pima Canyon, Yetman trail |
| Symphoricarpos occidentalis | Caprifoliaceae | Perennial herbaceous | root collar | 14 | Northern Great Plains |
| Symphyotrichum ascendens | Asteracceae | Perennial herbaceous | root | 4 | Wyoming Jackson |
| Symphyotrichum foliaceum | Asteraceae | Perennial herbaceous | root collar | 5 | Mt. Washburn |
| Thamnosma texana | Rutaceae | Perennial herbaceous | root | 30 | Saguaro East |
| Townsendia parryi | Asteraceae | Perennial herbaceous | root collar | 3 | Mt. Washburn |
| Tragopogon dubius | Asteraceae | Short-lived herbs | root | 1 | Fort Hays |
| Triodanis leptocarpa | Campanulaceae | Short-lived herbs | root | 1 | Fort Hays |
| Valeriana arizonica | Caprifoliaceae | Perennial herbaceous | root collar | 4 | Madera Canyon |
| Verbena bipinnatifida | Verbenaceae | Perennial herbaceous | root | 7 | Baboquivari, Fort Hays |
| Verbena bracteata | Verbenaceae | Perennial herbaceous | root collar | 7 | Wyoming Jackson |
| Verbena stricta | Verbenaceae | Perennial herbaceous | root | 13 | Fort Hays |
| Veronica americana | Plantaginaceae | Short-lived herbs | root | 1 | San Pedro River |
| Veronica wyomingensis | Plantaginaceae | Short-lived herbs | root | 1 | Mt. Washburn |
| Vicia americana ssp. minor | Fabaceae | Short-lived herbs | root | 1 | Fort Hays |
| Vitis arizonica | Vitaceae | Woody perennials | stem base | 8 | Pima Canyon |
| Xanthisma gracile | Asteraceae | Perennial herbaceous | root | 10 | Saguaro East |
| Zinnia acerosa | Asteraceae | Perennial herbaceous | root collar | 32 | Yetman trail |
| Zygophyllum californicum | Zygophyllaceae | Perennial herbaceous | root | 9 | Organ Pipe National Park |

**Table S3.** Summary of all models. “log” stands for natural logarithm, “CV” for coefficient of variation.

| **Response** | **Predictor/parameter** | **Estimate** | **F** | **P-value** | **Adj-R^2^** | **λ** |
| --- | --- | --- | --- | --- | --- | --- |
| log(Growth) | Intercept | 5.856 | - | - | 0.36 | 0.00 |
|  | Short-lived herbs | 1.209 | 57.572 | 0.000 |  |  |
|  | Woody perennials | -0.151 |  |  |  |  |
|  | log(NSC) | -0.074 | 1.275 | 0.260 |  |  |
| Growth CV | Intercept | 52.872 | - | - | 0.00 | 0.18 |
|  | Short-lived herbs | 8.649 | 1.447 | 0.239 |  |  |
|  | Woody perennials | 8.319 |  |  |  |  |
|  | log(NSC) | 1.149 | 0.170 | 0.681 |  |  |
| log(Longevity) | Intercept | 1.787 | - | - | 0.73 | 0.02 |
|  | Short-lived herbs | -1.619 | 276.279 | 0.000 |  |  |
|  | Woody perennials | 0.587 |  |  |  |  |
|  | log(NSC) | 0.045 | 1.169 | 0.281 |  |  |
| log(NSC) | Intercept | 1.830 | - | - | 0.05 | 0.06 |
|  | Short-lived herbs | -0.009 | 0.076 | 0.927 |  |  |
|  | Woody perennials | 0.073 |  |  |  |  |
|  | Temperature | 0.002 | 3.842 | 0.051 |  |  |
|  | Precipitation | 0.000 | 0.688 | 0.408 |  |  |
| log(Growth) | Intercept | 6.361 | - | - | 0.43 | 0.00 |
|  | Short-lived herbs | 0.972 | 40.673 | 0.000 |  |  |
|  | Woody perennials | -0.329 |  |  |  |  |
|  | log(NSC) | -0.151 | 5.579 | 0.019 |  |  |
|  | Temperature | 0.002 | 2.894 | 0.091 |  |  |
|  | Precipitation | -0.001 | 5.907 | 0.016 |  |  |
| Growth CV | Intercept | 56.931 | - | - | 0.10 | 0.15 |
|  | Short-lived herbs | 0.429 | 0.103 | 0.902 |  |  |
|  | Woody perennials | 2.961 |  |  |  |  |
|  | log(NSC) | -1.459 | 0.289 | 0.592 |  |  |
|  | Temperature | 0.095 | 6.218 | 0.014 |  |  |
|  | Precipitation | -0.017 | 1.104 | 0.295 |  |  |
| log(Longevity) | Intercept | 1.721 | - | - | 0.74 | 0.00 |
|  | Short-lived herbs | -1.671 | 261.086 | 0.000 |  |  |
|  | Woody perennials | 0.551 |  |  |  |  |
|  | log(NSC) | 0.029 | 0.461 | 0.498 |  |  |
|  | Temperature | 0.001 | 1.449 | 0.230 |  |  |
|  | Precipitation | 0.000 | 0.000 | 0.989 |  |  |
| log(Growth) | Intercept | 5.358 | - | - | 0.41 | 0.00 |
|  | Short-lived herbs | 1.109 | 49.527 | 0.000 |  |  |
|  | Woody perennials | -0.083 |  |  |  |  |
|  | log(Starch) | -0.060 | 9.586 | 0.002 |  |  |
|  | log(Fructans) | -0.056 | 5.170 | 0.024 |  |  |
|  | log(Simple sugars) | 0.282 | 11.718 | 0.001 |  |  |
| Growth CV | Intercept | 57.301 | - | - | -0.01 | 0.17 |
|  | Short-lived herbs | 9.592 | 1.607 | 0.204 |  |  |
|  | Woody perennials | 8.102 |  |  |  |  |
|  | log(Starch) | -0.095 | 0.013 | 0.911 |  |  |
|  | log(Fructans) | 0.432 | 0.082 | 0.775 |  |  |
|  | log(Simple sugars) | -2.207 | 0.371 | 0.543 |  |  |
| log(Longevity) | Intercept | 2.064 | - | - | 0.74 | 0.08 |
|  | Short-lived herbs | -1.557 | 246.746 | 0.000 |  |  |
|  | Woody perennials | 0.551 |  |  |  |  |
|  | log(Starch) | 0.017 | 1.668 | 0.198 |  |  |
|  | log(Fructans) | 0.043 | 7.099 | 0.008 |  |  |
|  | log(Simple sugars) | -0.143 | 6.809 | 0.010 |  |  |
| log(Starch) | Intercept | -2.989 | - | - | 0.03 | 0.44 |
|  | Short-lived herbs | -0.444 | 0.555 | 0.575 |  |  |
|  | Woody perennials | -0.278 |  |  |  |  |
|  | Temperature | 0.009 | 7.820 | 0.006 |  |  |
|  | Precipitation | 0.003 | 6.170 | 0.014 |  |  |
| log(Fructans) | Intercept | 2.272 | - | - | 0.08 | 0.09 |
|  | Short-lived herbs | -0.705 | 2.367 | 0.096 |  |  |
|  | Woody perennials | 0.127 |  |  |  |  |
|  | Temperature | -0.003 | 1.465 | 0.228 |  |  |
|  | Precipitation | -0.005 | 15.868 | 0.000 |  |  |
| log(Simple sugars) | Intercept | 1.342 | - | - | 0.03 | 0.31 |
|  | Short-lived herbs | 0.213 | 3.387 | 0.036 |  |  |
|  | Woody perennials | -0.187 |  |  |  |  |
|  | Temperature | 0.000 | 0.245 | 0.621 |  |  |
|  | Precipitation | 0.000 | 1.329 | 0.250 |  |  |
| log(Growth) | Intercept | 6.092 | - | - | 0.41 | 0.00 |
|  | Short-lived herbs | 0.972 | 40.075 | 0.000 |  |  |
|  | Woody perennials | -0.341 |  |  |  |  |
|  | Temperature | 0.001 | 1.856 | 0.175 |  |  |
|  | Precipitation | -0.001 | 5.157 | 0.024 |  |  |
| Growth CV | Intercept | 54.537 | - | - | 0.11 | 0.16 |
|  | Short-lived herbs | -0.085 | 0.099 | 0.906 |  |  |
|  | Woody perennials | 2.813 |  |  |  |  |
|  | Temperature | 0.091 | 5.951 | 0.016 |  |  |
|  | Precipitation | -0.017 | 1.092 | 0.298 |  |  |
| log(Longevity) | Intercept | 1.774 | - | - | 0.74 | 0.00 |
|  | Short-lived herbs | -1.671 |  |  |  |  |
|  | Woody perennials | 0.553 | 262.102 | 0.000 |  |  |
|  | Temperature | 0.001 | 1.732 | 0.190 |  |  |
|  | Precipitation | 0.000 | 0.001 | 0.980 |  |  |
| log(Growth) | Intercept | 5.651 | - | - | 0.48 | 0.00 |
|  | Short-lived herbs | 0.844 | 31.736 | 0.000 |  |  |
|  | Woody perennials | -0.275 |  |  |  |  |
|  | log(Starch) | -0.073 | 15.371 | 0.000 |  |  |
|  | log(Fructans) | -0.081 | 11.840 | 0.001 |  |  |
|  | log(Simple sugars) | 0.255 | 10.744 | 0.001 |  |  |
|  | Temperature | 0.002 | 4.491 | 0.035 |  |  |
|  | Precipitation | -0.001 | 5.135 | 0.025 |  |  |
| Growth CV | Intercept | 54.282 | - | - | 0.10 | 0.15 |
|  | Short-lived herbs | 0.408 | 0.074 | 0.929 |  |  |
|  | Woody perennials | 2.523 |  |  |  |  |
|  | log(Starch) | -0.679 | 0.667 | 0.415 |  |  |
|  | log(Fructans) | -0.449 | 0.096 | 0.757 |  |  |
|  | log(Simple sugars) | -1.556 | 0.203 | 0.653 |  |  |
|  | Temperature | 0.098 | 6.264 | 0.013 |  |  |
|  | Precipitation | -0.016 | 0.906 | 0.343 |  |  |
| log(Longevity) | Intercept | 1.96 | - | - | 0.74 | 0.05 |
|  | Short-lived herbs | -1.599 | 228.625 | 0.000 |  |  |
|  | Woody perennials | 0.530 |  |  |  |  |
|  | log(Starch) | 0.016 | 1.338 | 0.249 |  |  |
|  | log(Fructans) | 0.040 | 5.715 | 0.018 |  |  |
|  | log(Simple sugars) | -0.138 | 6.370 | 0.012 |  |  |
|  | Temperature | 0.001 | 0.951 | 0.331 |  |  |
|  | Precipitation | 0.000 | 0.022 | 0.883 |  |  |


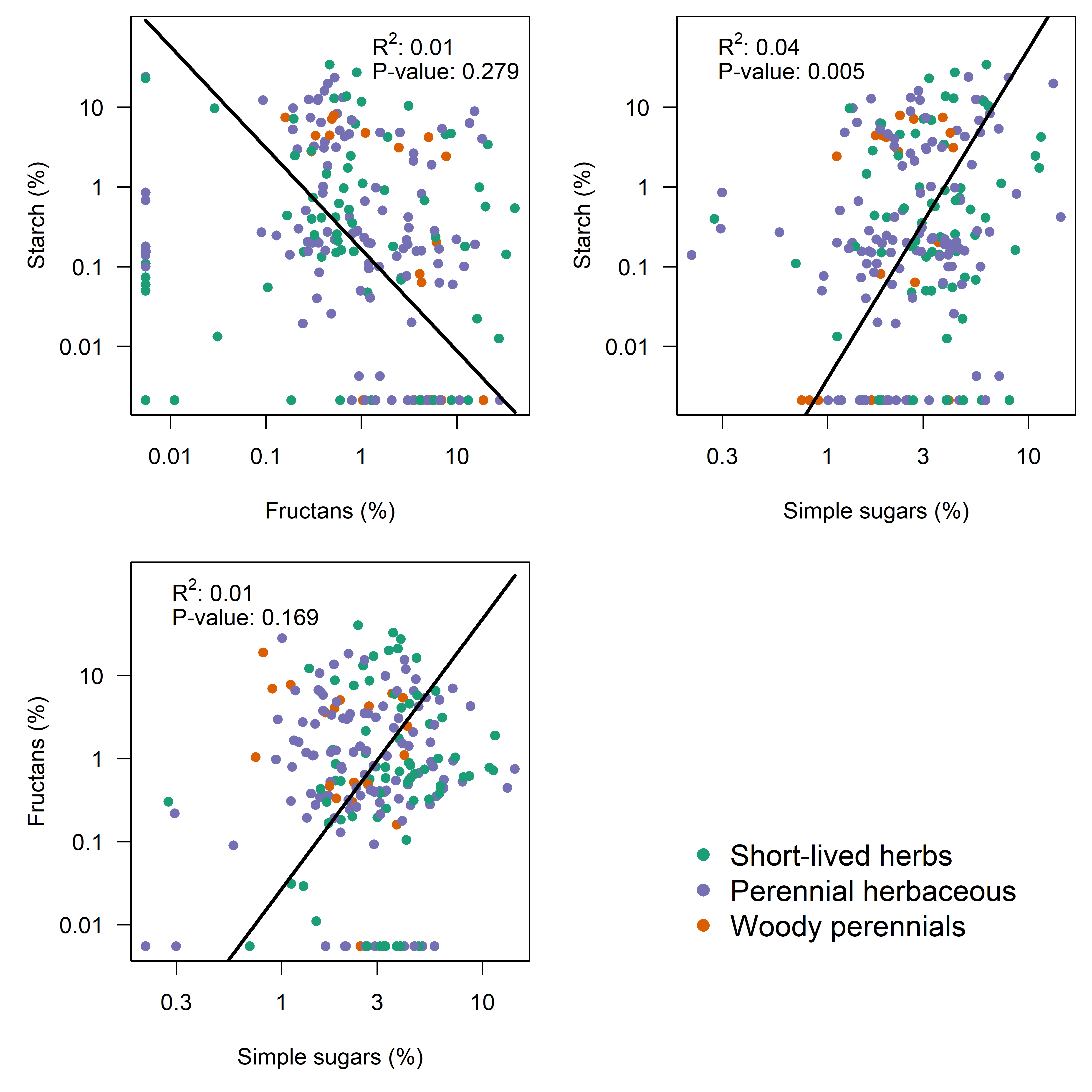


**Figure S1:** Standardized major axis of starch, fructans, and simple sugars (NSCs) of 201 species sampled at 43 locations across the Western United States. NSC values are expressed as a percentage of storage organ dry mass content.


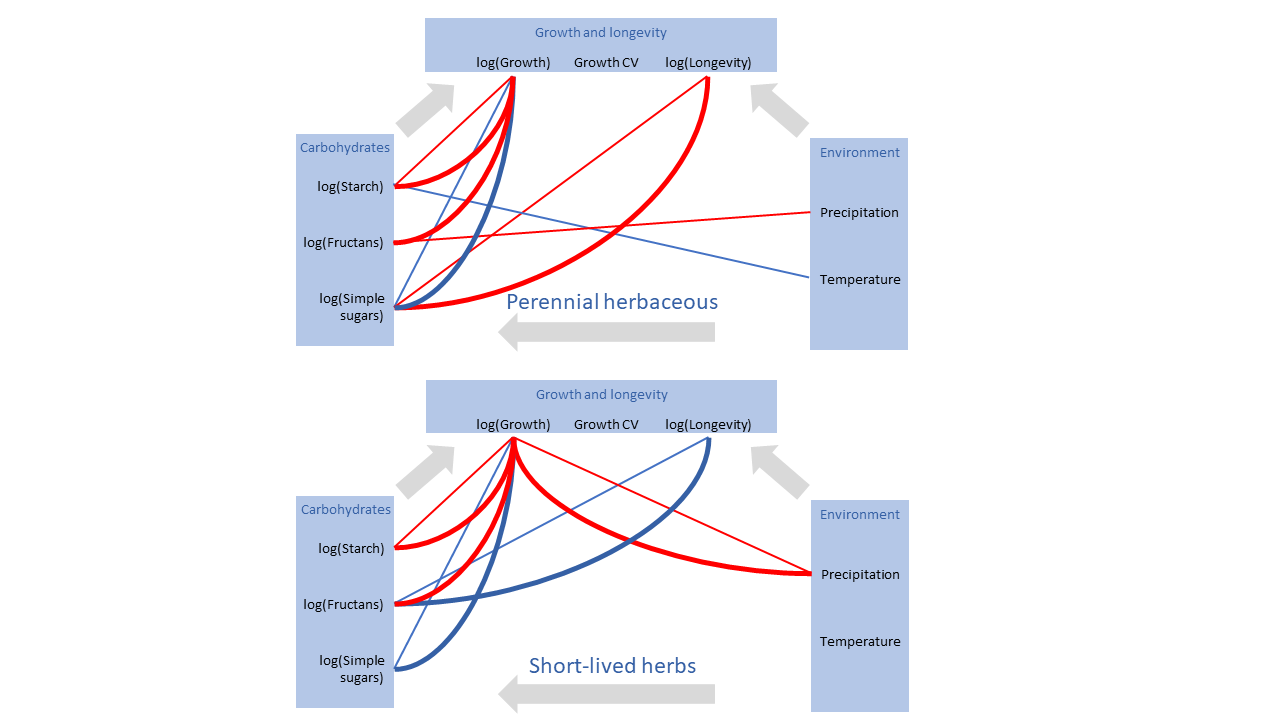


Figure S2: Links between environment, carbohydrates, and growth and longevity for herbaceous growth forms. Grey arrows show studied relationships. Red lines denote the negative effect, and blue lines positive effect. Curved lines are for analyses that take both carbohydrates and environmental variables into account. “log” stands for natural logarithm, and “CV” stands for coefficient of variation.
